# Radical‐Mediated Thiol‐Ene Strategy: Photoactivation of Thiol‐Containing Drugs in Cancer Cells

**DOI:** 10.1002/anie.201811338

**Published:** 2018-11-02

**Authors:** Shuang Sun, Bruno L. Oliveira, Gonzalo Jiménez‐Osés, Gonçalo J. L. Bernardes

**Affiliations:** ^1^ Department of Chemistry University of Cambridge Lensfield Road CB2 1EW Cambridge UK; ^2^ Departamento de Química. Centro de Investigación en Síntesis Química. Universidad de La Rioja 26006 Logroño Spain; ^3^ Instituto de Medicina Molecular Faculdade de Medicina Universidade de Lisboa Avenida Professor Egas Moniz 1649-028 Lisboa Portugal

**Keywords:** cage compounds, cancer, photochemistry, radicals, thiol-ene

## Abstract

Photoactivated drugs provide an opportunity to improve efficacy alongside reducing side‐effects in the treatment of severe diseases such as cancer. Described herein is a photoactivation decaging method of isobutylene‐caged thiols through a UV‐initiated thiol‐ene reaction. The method was demonstrated with an isobutylene‐caged cysteine, cyclic disulfide‐peptide, and thiol‐containing drug, all of which were rapidly and efficiently released under mild UV irradiation in the presence of thiol sources and a photoinitiator. Importantly, it is shown that the activity of histone deacetylase inhibitor largazole can be switched off when stapled, but selectively switched on within cancer cells when irradiated with non‐phototoxic light.

In recent decades, precision medicine has drawn a lot of attention for the effective treatment of various diseases, especially cancer. Currently, as a result of a lack of selectivity in the pathological sites, the development of new, effective, and safe therapies remains challenging. Among various new methods, the recently developed light‐mediated treatment is recognized as a promising approach to achieve controlled activation of medicine at pathological sites,^1^ and could significantly reduce the side effects of chemotherapy. So far, in the battle against cancer, several types of light‐activated anticancer reagents, which can be switched on conditionally with irradiation, have been investigated.^2^ The structures of these photocaged drugs include various ultraviolet, near‐infrared, or visible responsive moieties,^3^ such as *o*‐nitrobenzyl,2a, 4 coumarinyl ester,^5^ and metal complexes.2e, 6 These photoresponsive structures offer an extensive toolbox for use in cancer therapies and other biological applications. However, issues and challenges remain in this field, such as using nontoxic wavelengths, achieving rapid and efficient conversion, and improving the bioavailability of the prodrug. Therefore, there is still demand for new designs and new developments for photomediated therapy.

Thiol‐ene reactions (Scheme 1), a conjugation between a thiol and an alkene, have been known since the early 1900s.^7^ The coupling reaction proceeds through two mechanisms, namely photoinitiated free‐radical addition and catalyzed Michael addition reactions. There are several desirable features of a click reaction, including rapid reaction rates, ease of implementation, high yields and rates of conversion,^8^ so thiol‐ene “click” reactions have been increasingly used for various applications, such as biofunctionalization,^9^ surface and polymer modification, polymerization,^10^ and so on.^8^, ^11^ Recently, isobutylene‐bridged polymer networks have been extensively studied to synthesize polymer networks through radical‐initiated thiol‐ene chemistry.^12^ This covalently cross‐linked network is able to undergo photomediated, reversible cleavage of its isobutylene backbone to allow chain rearrangement and relieve structural strain. This method has also been used to provide a reactive handle for reversible addition and exchange of biochemical moieties under cytocompatible conditions.12d Key to this reaction is the isobutylene structure capable of addition‐fragmentation chain transfer (AFCT), in which the structure is attacked by the photoinitiated thiol radical in the presence of a photoinitiator (PI) to release the caged thiol part.

**Scheme 1:**
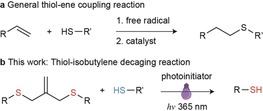
a) General thiol‐ene coupling reaction. b) The thiol‐isobutylene decaging reaction.

Inspired by this AFCT reaction, we hypothesized that the the isobutylene structure could be used as a bridging graft to cage thiol‐containing drugs and allow further controlled activation of anticancer drugs by means of a radical‐mediated thiol‐ene mechanism (Scheme 1). Previously, we reported a one‐pot macrocyclization strategy [with tris(2‐carboxyethyl)phosphine (TCEP)] for thiol‐containing peptides by using an isobutylene graft, which can significantly enhance both membrane permeability and binding activity of the corresponding macrocycles.^13^ The isobutylene graft can be rapidly and efficiently installed onto reduced thiol groups in a biocompatible manner because of the high reactivity of the bis(bromo)isobutylene. Our research began with *N*‐*tert*‐butoxycarbonyl‐l‐cysteine methyl ester (*N*‐Boc‐Cys‐OMe **1**), which was protected with 3‐bromo‐2‐bromomethyl‐1‐propene (Table 1 and Supporting Information). The stapled cysteine **2** was then screened under a series of reaction conditions. Firstly, different PIs were tested with the same amounts of thiol source, 1‐thio‐β‐d‐glucose tetraacetate (4AcGlcSH), and **2** under irradiation at 365 nm. The reaction with the water‐soluble PI 2,2′‐azobis[2‐(2‐imidazolin‐2‐yl)propane]dihydrochloride (Vazo 44) did not progress after two hours. However, the other PI, 2,2‐dimethoxy‐2‐phenylacetophenone (DPAP) successfully promoted the reaction and released **1**. β‐Mercaptoethanol (BME) was also tested as the thiol compound under the same reaction conditions. This reaction released the cysteine in a slightly lower yield, which suggests that different thiol sources could be used to promote the reaction. However, when *N*,*N*‐dimethylformamide (DMF) was used as solvent, two mixed disulfides resulting from the reaction between the 1‐thioglucose and *N*‐Boc‐cysteine methyl ester **1**′ were observed. To evaluate the reaction and calculate the isolated yield, TCEP was added to reduce the disulfides after the reaction. Under these reaction conditions, the reaction was complete within 15 minutes and gave a relatively high yield (65 %). Moreover, the reaction was also rapid and efficient when glutathione was used as a thiol source in a mixture of DMF and water as the solvent, which shows that the reaction is practical for an aqueous environment. Then, the feasibility of our strategy was investigated with an isobutylene cyclized 5‐mer peptide that bears two terminal cysteines and was synthesized by solid‐phase peptide synthesis.^13^ The short peptide was completely converted into the disulfide derivative within 15 minutes under the same reaction conditions described previously (Figure 1).

**Figure 1:**
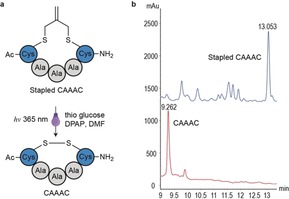
Thiol‐ene decaging reaction of an isobutylene‐cyclized peptide. a) The decaging reaction of stapled CAAAC peptide with 1‐thio‐β‐d‐glucose tetraacetate. b) HPLC traces of the starting material and reaction mixture. Blue: stapled CAAAC peptide; red: reaction mixture after 15 min.

**Table 1:** Optimization of the thiol‐ene decaging reaction. 
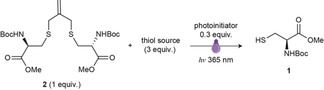

Entry	PI	Thiol	Solvent	TCEP	*t* [min]	Yield [%]
1	Vazo44	4AcGlcSH	MeOH	−	120	0
2	DPAP	4AcGlcSH	CH_2_Cl_2_	−	120	54
3	DPAP	BME	CH_2_Cl_2_	−	120	37
4	DPAP	4AcGlcSH	DMF	−	120	^[a]^
5	DPAP	4AcGlcSH	DMF	−	15	^[a]^
6	DPAP	4AcGlcSH	DMF	+	15	65
7	DPAP	GSH	DMF/H_2_O 1:1	+	15	67

[a] A mixture of disulfides formed by reaction of cysteine and 1‐thioglucose and two cysteines. GSH=glutathione; PI=photoinitiator.

To demonstrate that the release of the thiol compound occurs by means of a radical‐mediated thiol‐ene mechanism, a series of control experiments were conducted (Table 2 and Supporting Information). As shown in Table 2, UV irradiation (entry 2), a thiol source (entry 3), and a PI are essential for the decaging reaction. When the radical scavenger (2,2,6,6‐tetramethylpiperidin‐1‐yl)oxyl (TEMPO) was added to the reaction, it terminated the reaction by forming an intermediate with the DPAP fragmentation radical, which suggests that the reaction is mediated by radicals.

**Table 2:** Control studies of thiol‐ene decaging reactions between isobutylene‐grafted cysteine and thiol‐glucose.

Entry	Thiol	Stapled Cys **2**	UV	DPAP	TEMPO	Conversion [%]
1	+	+	+	+	−	100
2	+	+	−	+	−	no reaction
3	+	−	+	+	−	no reaction
4	−	+	+	+	−	no reaction
5^[a]^	+	+	+	+	+	no reaction

[a] The formation of the intermediate, 2,2,6,6‐tetramethylpiperidin‐1‐yl benzoate, by the reaction between TEMPO and DPAP was monitored by MS‐ESI^+^.

The proposed mechanism for the radical‐mediated thiol‐ene decaging reaction was studied by quantum mechanical calculations using abbreviated thiol models (see the Supporting Information) and is shown as Scheme 2. After generation of the thiol radical by the PI under UV irradiation, the isobutylene grafted structure undergoes a fast thiol‐ene anti‐Markovnikov addition with a calculated activation energy of Δ*G* ^≠^≈14 kcal mol^−1^ at the PCM(H_2_O)/M06‐2X/6‐31++ G(2,p) theory level, to generate a symmetric tertiary‐carbon‐centered radical intermediate. Then, the unstable radical intermediate undergoes a β‐scission at a very similar reaction rate, regenerating the isobutylene linkage and resulting in a mixed caged compound, which can be again attacked by another thiol radical following the same mechanism to release the other unit of the caged thiol compound. The process is nearly thermoneutral and reversible until two decaged radical thiols collapse forming a stable disulfide bond (Scheme 2).

**Scheme 2:**
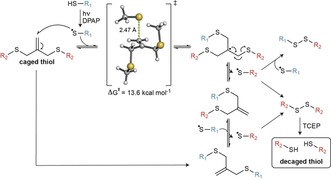
Proposed mechanism for the radical‐mediated thiol‐isobutylene decaging reaction.

With all this knowledge in hand and to demonstrate that this strategy is practical to activate drugs in vitro, we applied our method to the potent histone deacetylase inhibitor (HDAC) largazole. The cyclic depsipeptide largazole is a marine natural product, and its derivatives are recognized as promising potential anticancer therapeutics. Largazole possesses remarkable and preferential growth‐inhibitory activity against cancer cell lines relative to corresponding nontransformed cells.^14^ The octanoyl tail in largazole has better cell permeability than the active free thiol species, largazole thiol, the latter being formed inside cells by esterase or lipase‐based cleavage of the octanoyl residue.^15^ The free thiol group binds to the active site Zn^2+^‐domain within the HDAC enzyme, and results in a potent inhibitory effect. Thus, the thiol‐ene decaging strategy can be used to protect the thiol group, improve the cell‐permeability, and allow controlled activation with UV. Therefore, we synthesized three largazole derivatives: largazole, largazole thiol, and stapled largazole (see the Supporting Information). The result of the parallel artificial membrane permeability assay indicated that stapled largazole is a highly passively permeable compound (logP_e_ −5.29 and P_e_ 5.3×10^−6^ cm sec^−1^; see Table S4 in the Supporting Information). Then, the stapled largazole was reacted with 1‐thio‐β‐d‐glucose tetraacetate and DPAP under UV irradiation for 15 minutes (Figure 2 a). Full conversion of the stapled largazole was observed in the HPLC trace along with the appearance of a largazole thiol signal (Figure 2 b). Next, the growth‐inhibitory activity was evaluated with human colon carcinoma cell lines, HCT‐116 (Figure 2 c). As expected,^16^ largazole (GI_50_ 1.433 nm) is more potent than the corresponding free thiol species (GI_50_ 185.1 nm) owing to its octanoyl side‐chain which improves cell permeability and allows facile presentation of the free thiol within the cell.^15^ The stapled largazole (GI_50_ 407.7 nm) is a much less potent compound because the thiol group is protected by the isobutylene structure, which prevents binding with the Zn^2+^ domain in cells. Before testing the decaging conditions in cells, we investigated the toxicity of DPAP and phototoxicity of the light in terms of the power and the irradiation time (see Figure S7). A set of cytocompatible conditions, 15 minute irradiation at 80 W, were chosen to conduct further investigations.

**Figure 2:**
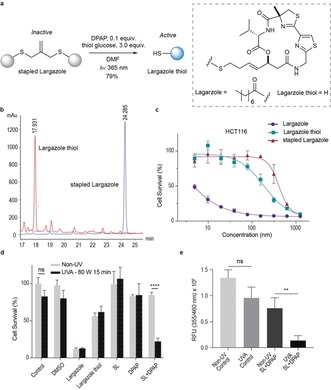
The photoactivation of isobutylene‐grafted largazole thiol. a) Thiol‐ene decaging reaction of stapled largazole with 1‐thio‐β‐d‐glucose tetraacetate. HPLC traces of the reaction mixture. Blue: stapled largazole; red: reaction mixture after 15 min. b) Growth inhibitory effects of largazole, largazole thiol, and stapled largazole on HCT‐116 colon carcinoma cells: for largazole GI_50_ 1.433 nm, for largazole thiol GI_50_ 185.1 nm, and for stapled largazole GI_50_ 407.7 nm. c) Cell survival under different conditions. SL=stapled largazole, UV condition, 365 nm 80 W, 15 min. d) HDAC activity assay of control group and UV group. Data are representative of three independent tests and analyzed by the two‐tailed unpaired Student's t‐test. **, P<0.01; ****, P<0.0001. Error bars reflect one standard deviation from the mean.

The decaging reaction of stapled largazole was tested with HCT‐116 cells at 150 nm. McCoy's 5A culturing medium contains cysteine and glutathione, so no other thiol source was added to the medium. The cell viabilities of the three drug groups with/without UV irradiation were consistent with the growth‐inhibitory assay (Figure 2 d). The UV‐irradiated premixed group of stapled largazole and DPAP showed significantly lower cell viability than the corresponding non‐irradiation group, the stapled largazole group, and the largazole thiol group. To confirm the results, a fluorometric HDAC activity assay was conducted with HCT‐116 cell lysates (Figure 2 e). A significant decrease of fluorescence was observed in the UV‐irradiated premixed group relative to the control groups and the non‐irradiation group. Both the cell viability and the enzyme activity results indicated that the stapled largazole was successfully activated by UV light.

In summary, we have developed a rapid and efficient thiol‐ene‐based photoactivation strategy for thiol‐containing drugs caged using isobutylene. The radical‐mediated reaction, that is triggered by UV light, undergoes a thiol‐ene addition step to form an unstable radical intermediate which is further cleaved by β‐scission to release the caged thiols. We applied this method to various substrates, such as *N*‐Boc‐cysteine, a cysteine‐containing peptide, and the HDAC inhibitor largazole, and showed that the caged thiol molecules, unlike their free counterparts, display high membrane permeability. The successful activation of largazole in HCT‐116 cells demonstrates the potential as a drug delivery and activation method for cancer therapy. In vivo, this strategy may explore radical initiators that are often characteristic of cancer cells to achieve targeted drug activation. And finally, further investigation of the use of this strategy on proteins could also be useful for photocontrolled protein activation and drug release from antibody–drug conjugates.

## Conflict of interest

The authors declare no conflict of interest.

## Supporting information

As a service to our authors and readers, this journal provides supporting information supplied by the authors. Such materials are peer reviewed and may be re‐organized for online delivery, but are not copy‐edited or typeset. Technical support issues arising from supporting information (other than missing files) should be addressed to the authors.

Supplementary
