# Supplementary material for: Radical‐Mediated Thiol‐Ene Strategy: Photoactivation of Thiol‐Containing Drugs in Cancer Cells
Source: Angew Chem Int Ed Engl. 2018 Nov 2;57(48):15832–5. doi: 10.1002/anie.201811338 (PMC6391964; doi:10.1002/anie.201811338)
Supplement: Supplementary file 1 — Supplementary [file ANIE-57-15832-s001.pdf]

Angewandte Chemie
Eine Zeitschrift der Gesellschaft Deutscher Chemiker

Supporting Information

Radical-Mediated Thiol-Ene Strategy: Photoactivation of Thiol-Containing Drugs in Cancer Cells

*Shuang Sun, Bruno L. Oliveira, Gonzalo Jiménez-Osés, and Gonçalo J. L. Bernardes**

anie_201811338_sm_miscellaneous_information.pdf

Supporting Information

Table of Contents

Experimental Section 2

General methods 2

Unstapling the stapled cysteine 2 2

Unstapling short peptide CAAAC 3

Largazole synthesis and decaging 4

Computational Details 19

Biological Assays 22

Parallel Artificial Membrane Permeability Assay (PAMPA) 22

Cell Culture 23

Cell viability assay 23

UV Condition Screening 24

Photoactivation of Stapled Largazole in HCT-116 cells 24

Histone Deacetylase (HDAC) Activity Assay (Fluorometric) 24

NMR Spectrum 26

References 38

Experimental Section

General methods

Chromatography. Analytical thin-layer chromatography (TLC) was carried out on Merck silica gel 60 F254 plates, using UV at 254 nm or staining with ninhydrin for visualization. Column chromatography was performed with Material Harvest silica gel 60. Reverse-phase column chromatography was conducted with Varian Bond Elut® C18. The HPLC was conducted on Agilent 1100 Series fitted with G1322A degasser, G1311A pump, G1313A autosampler and G1315 DAD, with YMC-Pack Pro C18 column 120 Å S-5 µm 10 mm x 250 mm (product no. AS12S05-2510WT) for preparative scale. The eluent was solvent B, water with 0.1% trifluoroacetic acid (TFA), and C, acetonitrile with 0.1% trifluoroacetic acid, unless otherwise noted and gradients specific to the compound.

Characterization. ^{1}H NMR was recorded on Bruker 400-Avance III, DPX-400 or 500-DCH Cryoprobe as appropriate. ^{13}C NMR were recorded by the Department of Chemistry NMR service. Chemical shifts (ppm) were referenced to the residual proton signal of the solvent. High resolution mass spectra were obtained by the Department of Chemistry Mass spectrometry service with a Thermo Fischer LTQ Orbitrap Discovery and ionised by electrospray (ESI).

Unstapling the stapled cysteine 2

The stapled cysteine **2** was synthesised following the previously reported method[1]. DPAP (0.006 mmol, 0.3 equiv) and thiol reagents (0.06 mmol, 3.0 equiv) was dissolved to a solution of compound **2** (0.02 mmol, 1.0 equiv) in DMF. After being degassed with Ar, the solution was irradiated with in the UV reactor (LZC-ORG, fitted with 10 8W UVA lamps). When the TLC indicated the disappearance of starting material, the reaction was removed from the reactor. After being stirred in air for one hour, TCEP (0.04 mmol, 2.0 equiv) was added into the solution. After 1 h, the mixture was concentrated under reduced pressure and purified by column chromatography (petrol/ethyl acetate, 3:1) to provide the free cysteine **1** as white solid.

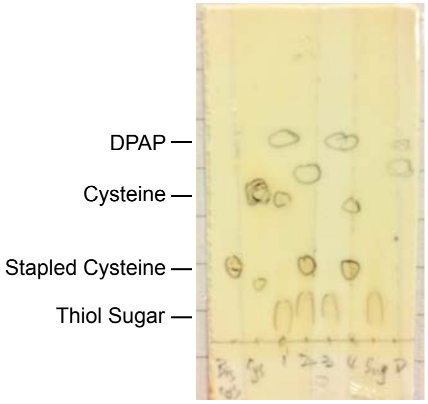

Figure S1. Thin-layer chromatography (TLC) of control studies.

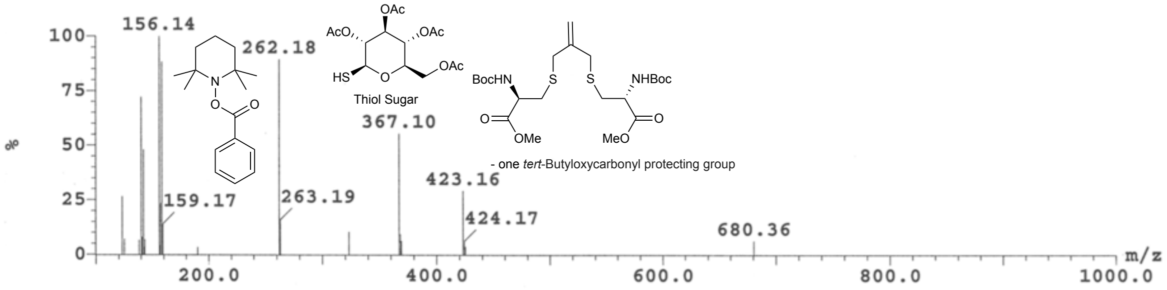

Mass	Calc. Mass	mDa	PPM	DBE	Formula	i-FIT
262.1802	262.1807	-0.5	-1.9	5.5	C16 H24 N O2	19.6
262.1802	262.1794	0.8	3.1	6.0	C14 H22 N4 O	28.7
262.1802	262.1812	-1.0	-3.8	-1.5	C H20 N13 O3	2876.1

Figure S2. MS-ESI+ for TEMPO control experiment.

Unstapling short peptide CAAAC

Short peptide CAAAC was synthesised with solid-phase peptide synthesis method following previously reported method[1]. DPAP (0.6 mg, 2.4 µmol 1.0 equiv) and 1-thio-*β*-D-glucose tetraacetate, 4AcGlcSH, (8.9 mg, 0.024 mmol, 10.0 equiv) was dissolved to a solution of stapled CAAAC **5** (1.3 mg, 2.4 µmol, 1.0 equiv) in DMF. The solution was degassed with Ar. Then the UV light was turned on. When the LC-MS indicated the disappearance of starting material, the UV light was turned off. After being stirred in air for one hour, TCEP (4.2 mg, 0.015 mmol, 6.0 equiv) was added to the solution. After 1 h, the solvent was removed by air flush and purified by HPLC. HRMS (ESI+) m/z: calcd. for $C_{17}H_{28}N_6O_6S_2Na$ $[M+Na]^+$: 499.1404 found: 499.1401.

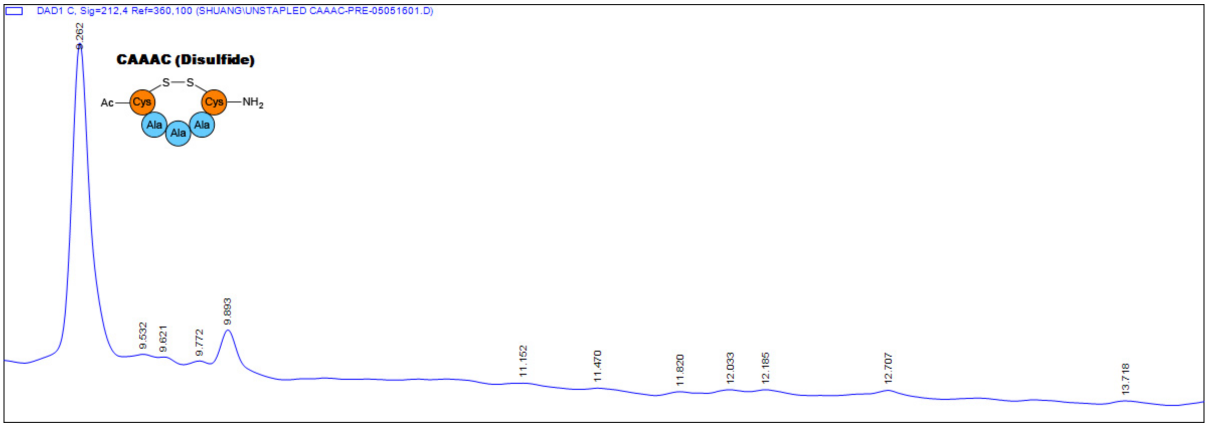

Figure S3. HPLC chromatogram of unstapled short peptide.

Table S1. The gradient of eluent for peptide **CAAAC**.

Solvents	0 min	20 min	22 min	27 min	29 min
B	95%	38.3%	10%	95%	95%
C	5%	61.7%	90%	5%	5%

Flow rate: 3.0 mL/min

Largazole synthesis and decaging

The Largazole thiol was synthesised following several reported routes. (Scheme 1-4)

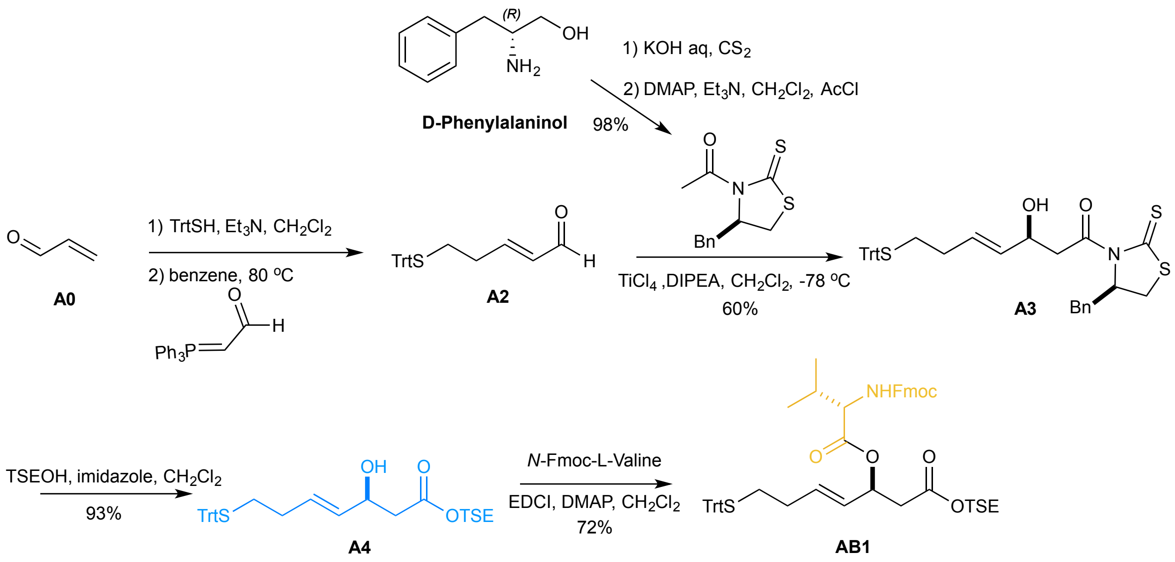

Scheme S1. Synthesis of building block A and conjugation with *N*-Fmoc-L-Valine.

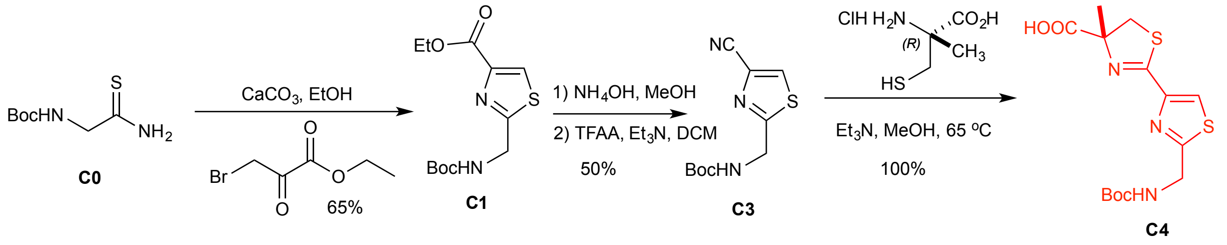

Scheme S2. Synthesis of building block C.

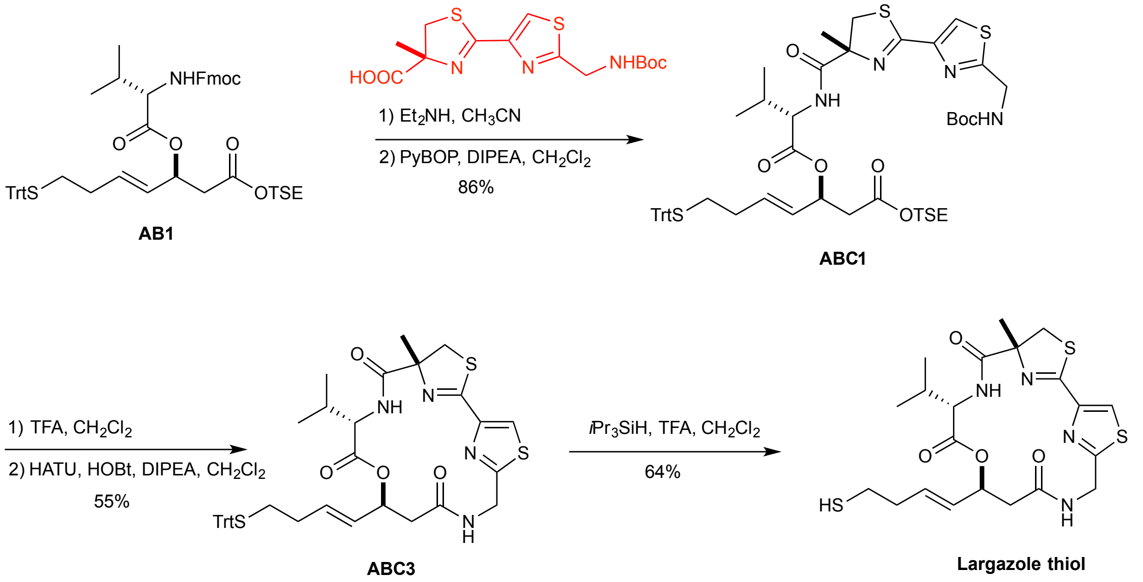

Scheme S3. Conjugation of building block AB with C, macrocyclization and deprotection.

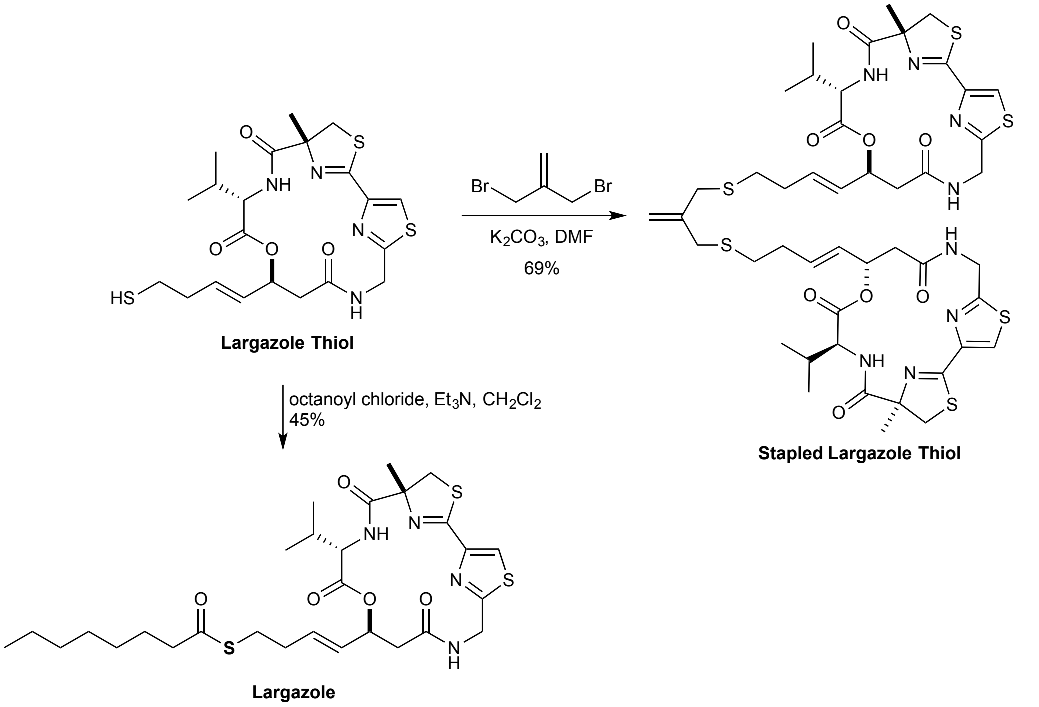

Scheme S4. Synthesis of Largazole and stapling of Largazole Thiol.

*Synthesis of ethyl 2-[N-(tert-butoxycarbonyl)aminomethyl]thiazole-4-carboxylate (**C1**)*

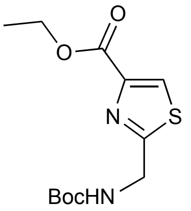

Ethyl bromopyruvate (0.84 mL, 6.7 mmol, 1.2 equiv) and calcium carbonate (0.56 g, 5.6 mmol, 1 equiv) were added in sequence to a solution of *tert*-butyl 2-amino-2- thioxoethylcarbamate **C0** (1.06 g, 5.6 mmol, 1 equiv) in ethanol (20 mL) at room temperature. The reaction mixture was stirred for 7 h at room temperature. The product mixture was concentrated, and the residue obtained was purified by flash-column chromatography (petroleum ether 40-60/ethyl acetate, 8:1 to 2:1) to furnish the thiazole **C1** as a white solid (1.02 g, 65%).

^{1}H NMR (400 MHz, CDCl$_3$) δ 8.11 (s, 1H), 4.64 (d, *J* = 6.3 Hz, 2H), 4.41 (q, *J* = 7.1 Hz, 2H), 1.45 (s, 9H), 1.39 (t, *J* = 7.1 Hz, 3H); ^{13}C NMR (100 MHz, CDCl$_3$) δ 170.09, 161.43, 155.73, 147.07, 128.03, 80.63, 61.67, 42.53, 28.45, 14.52.

The preparation was followed the reference and the characterization was in agreement with those reported[2].

*Synthesis of 2-[N-(tert-butoxycarbonyl)aminomethyl]thiazole-4-carboxamide (**C2**)*

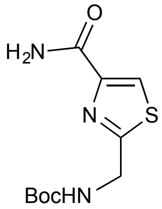

A solution of aqueous ammonia (28% w/v, 42 mL) was added to a solution of the thiazole **C1** (1.01 g, 3.5 mmol, 1 equiv) in anhydrous methanol (35 mL) at room temperature. The resulting mixture was stirred overnight at room temperature. The product mixture was concentrated and the residue obtained was dried by azeotropic distillation from toluene (50 mL*2) to afford the product **C2** as a yellow solid (0.87 g, 96%).

^{1}H NMR (400 MHz, $CDCl_3$) δ 8.09 (s, 1H), 7.11 (s, 1H), 5.74 (s, 1H), 5.27 (s, 1H), 4.60 (d, *J* = 6.2 Hz, 2H), 1.47 (s, 9H); ^{13}C NMR (100 MHz, DMSO) δ 171.66, 162.42, 155.92, 150.07, 124.17, 78.88, 42.08, 28.29.

The preparation was followed the reference and the characterization was in agreement with those reported[2].

*Synthesis of 2-[N-(tert-butoxycarbonyl)aminomethyl]thiazole-4-carbonitrile (**C3**)*

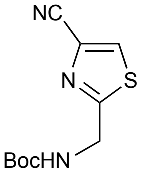

Trifluoroacetic anhydride (0.51 mL, 3.6 mmol, 1.1 equiv) was added dropwise over 20 min to a solution of the amide **C1** (0.086 g, 3.3 mmol, 1 equiv) and triethylamine (1.0 mL, 7.26 mmol, 2.20 equiv) in dichloromethane (50 mL) at 0 °C. The resulting mixture was stirred for 30 min at 0 °C. The reaction mixture was then allowed to warm over 30 min to room temperature. The reaction mixture was stirred for 2 h at room temperature. The product mixture was concentrated, and the

residue obtained was purified by flash-column chromatography (petroleum ether 40-60/ethyl acetate, 4:1 to 3:1) to furnish the nitrile **C3** as a white solid (0.4 g, 50%).

^{1}H NMR (500 MHz, $CDCl_3$) δ 7.94 (s, 1H), 5.43 – 5.19 (m, 1H), 4.62 (d, *J* = 6.3 Hz, 2H), 1.47 (s, 9H); ^{13}C NMR (125 MHz, $CDCl_3$) δ 171.39, 155.61, 130.84, 126.49, 113.79, 80.85, 42.33, 28.28.

The preparation was followed the reference and the characterization was in agreement with those reported[2].

*Synthesis of (R)-2-(2-((tert-butoxycarbonylamino)methyl)thiazol-4-yl)-4-methyl-4,5-dihydrothiazole-4-carboxylic acid (**C4**)*

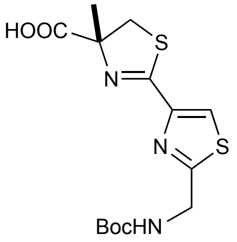

Triethylamine (0.37 mL, 2.65 mmol, 1.6 equiv) was added dropwise to a solution of the nitrile **C3** (395.6 mg, 1.65 mmol, 1 equiv) and 2-methyl-L-cysteine (454.0 mg,2.65 mmol, 1.6 equiv) in methanol (25 mL) at room temperature. The reaction mixture was heated at reflux overnight. The product mixture was cooled to room temperature and the cooled product mixture was concentrated. The residue obtained was dissolved in saturated aqueous sodium bicarbonate solution (40 mL) and the resulting solution was washed with ether (30 mL). The aqueous layer was acidified to pH 3~4 by the dropwise addition of 3.0 N aqueous hydrochloric acid solution. The resulting mixture was extracted with ethyl acetate (3*30 mL) and the organic layers were combined. The combined organic layers were dried over sodium sulfate and the dried solution was filtered. The filtrate was concentrated to provide the thiazole–thiazoline **C4** as a white solid (590.9 mg, 100%).

^{1}H NMR (400 MHz, $CDCl_3$) δ 7.95 (s, 1H), 5.32 (s, 1H), 4.63 (d, *J* = 6.3 Hz, 2H), 3.86 (d, *J* = 11.6 Hz, 1H), 3.36 (d, *J* = 11.6 Hz, 1H), 1.67 (s, 3H), 1.47 (s, 9H); ^{13}C NMR (100 MHz, $CDCl_3$) δ 174.98, 170.12, 164.57, 155.63, 147.93, 122.70, 84.51, 80.57, 42.29, 40.97, 28.32, 24.33.

The preparation was followed the reference and the characterization was in agreement with those reported[3].

*Synthesis of (R)-1-(4-benzyl-2-thioxothiazolidin-3-yl)ethanone (**Auxiliary**)*

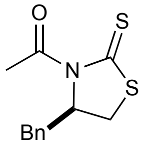

D-Phenylalaninol (2.5 g, 16.5 mmol, 1 equiv) was dissolved in aqueous KOH (3 M, 35 mL). CS_2 (5.0 mL, 82.7 mmol, 5 equiv) was added and the solution was heated at reflux overnight. The solution was extracted with dichloromethane (3*200 mL), dried (Na_2SO_4), filtered, and concentrated to afford (*R*)-4-benzylthiazolidine-2-thione, which was used without further purification. The auxiliary (16.5 mmol, 1 equiv), 4-dimethylaminopyridine (0.2 g, 1.65 mmol, 0.1 equiv), and triethylamine (3.4 mL, 24.8 mmol, 1.5 equiv) were dissolved in dry CH_2Cl_2 (45 mL) and cooled to 0 ^oC. Acetyl chloride (1.8 mL, 24.8 mmol, 1.5 equiv) was added dropwise and the reaction was allowed to reach room temperature and stirred overnight. Then, the reaction was quenched with satd NH_4Cl (48 mL), diluted with Et_2O (45 mL), and the organic phase was washed with satd $CuSO_4$ (3*20 mL), water (20 mL), and brine (20 mL), dried (MgSO4), filtered, and concentrated to give the crude compound as a yellow solid. Recrystallization from EtOH afforded the title **Auxiliary** (4.0 g, 98%) as yellow needles

^1H NMR (500 MHz, $CDCl_3$) δ 7.36 – 7.26 (m, 5H), 5.41 – 5.34 (m, 1H), 3.38 (ddd, *J* = 11.6, 7.3, 1.1 Hz, 1H), 3.21 (dd, *J* = 13.2, 3.8 Hz, 1H), 3.03 (dd, *J* = 13.2, 10.6 Hz, 1H), 2.88 (dd, *J* = 11.5, 0.7 Hz, 1H), 2.79 (s, 3H); ^{13}C NMR (125 MHz, $CDCl_3$) δ 201.71, 170.86, 136.65, 129.59, 129.05, 127.37, 68.36, 36.83, 31.96, 27.22.

The preparation was followed the reference and the characterization was in agreement with those reported[4].

*Synthesis of (2E)-5-[(triphenylmethyl)thio]-2-pentenal (**A2**)*

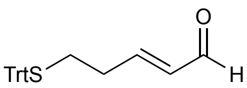

To a solution of triphenylmethanethiol (2.7 g, 9.8 mmol, 2.09 equiv) in dichloromethane (100 mL) was added acrolein **A0** (0.8 g, 13.6 mmol, 2.9 equiv) and triethylamine (1.4 g, 13.6 mmol, 2.9 equiv). The resulting mixture was stirred for 1 h at room temperature and was concentrated to give the aldehyde **A1** as a white solid, which was used in the next step without purification. A

solution of the aldehyde **A1** obtained above and (tri- phenylphosphoranylidene)acetaldehyde (1.4 g, 4.7 mmol, 1 equiv) in dry benzene (58 mL) was refluxed overnight. The reaction mixture was concentrated and purified by flash-column chromatography (petroleum ether 40-60/dichloromethane, 1:3 to 1:1) to afford aldehyde **A2** (1.68 g, 100%).

^{1}H NMR (400 MHz, $CDCl_3$) δ 9.43 (d, *J* = 7.8 Hz, 1H), 7.45 (dd, *J* = 7.8, 1.8 Hz, 6H), 7.30 (dd, *J* = 8.5, 6.7 Hz, 6H), 7.26 – 7.20 (m, 3H), 6.63 (dt, *J* = 15.6, 6.4 Hz, 1H), 5.99 (ddt, *J* = 15.7, 7.9, 1.4 Hz, 1H), 2.34 (dtd, *J* = 22.3, 6.5, 2.0 Hz, 4H); ^{13}C NMR (100 MHz, $CDCl_3$) δ 193.82, 155.87, 144.63, 133.67, 129.59, 128.05, 128.00, 127.97, 126.86, 67.06, 31.79, 30.08.

The preparation was followed the reference and the characterization was in agreement with those reported[5].

*Synthesis of 3S-hydroxy-1-(4R-benzyl-2-thioxothiazolidin-3-yl)-7-tritylsulfanylhept-4E-en-1-one (**A3**)*

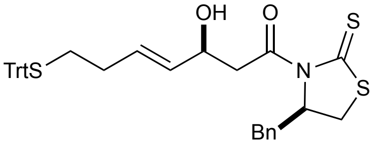

To a solution of acetyl Nagao chiral auxiliary (2.07 g, 8.24 mmol, 1 equiv) in dichloromethane (42 mL) at 0 °C was added $TiCl_4$ (1.0 mL, 9.04 mmol, 1.23 equiv). After the reaction mixture was stirred for 5 min and cooled to 78 °C, DIPEA (1.6 m, 9.06 mmol, 1.25 equiv) was added. The mixture was stirred for 2 h at the same temperature, and to it was added the aldehyde **A2** (1.74 g, 4.85 mmol, 0.987 equiv) in dichloromethane (5 mL) dropwise. The reaction mixture was stirred for 1 h at 78 °C. It was removed from cooling bath, treated with water (15 mL), and diluted with dichloromethane (50 mL). The aqueous portion was extracted with dichloromethane; the organic layer was washed with saturated NaCl (40 mL) and dried over anhydrous Na_2SO_4. It was concentrated in vacuo and the residue was purified by flash-column chromatography (petroleum ether 40-60/dichloromethane, 1:2 to 1:3) to obtain the compound **A3** (1.79 g, 60%) as a thick yellow oil.

^{1}H NMR (500 MHz, $CDCl_3$) δ 7.43 – 7.38 (m, 6H), 7.37 – 7.32 (m, 2H), 7.31 – 7.27 (m, 9H), 7.23 – 7.16 (m, 3H), 5.60 (dtd, *J* = 15.5, 6.6, 1.2 Hz, 1H), 5.48 (ddt, *J* = 15.5, 6.0, 1.3 Hz, 1H), 5.36 (ddd, *J* = 10.7, 7.0, 3.9 Hz, 1H), 4.63 – 4.55 (m, 1H), 3.56 (dd, *J* = 17.6, 3.0 Hz, 1H), 3.37 – 3.26 (m, 2H),

3.21 (dd, *J* = 13.2, 3.9 Hz, 1H), 3.03 (dd, *J* = 13.1, 10.5 Hz, 1H), 2.86 (d, *J* = 11.6 Hz, 1H), 2.69 (d, *J* = 4.6 Hz, 1H), 2.22 (dd, *J* = 7.7, 6.2 Hz, 2H), 2.10 (q, *J* = 7.3 Hz, 2H); ^{13}C NMR (125 MHz, $CDCl_3$) δ 201.29, 172.55, 144.85, 136.37, 131.90, 130.09, 129.63, 129.57, 129.43, 128.93, 127.85, 127.28, 126.58, 68.39, 68.33, 66.56, 45.58, 36.75, 32.07, 31.44, 31.35.

The preparation was followed the reference and the characterization was in agreement with those reported[5].

*Synthesis of (3S,4E)-3-hydroxy-7-[(triphenylmethyl)thio]-4-heptenoic acid (2-trimethylsilyl)ethyl ester (**A4**)*

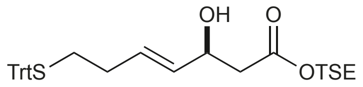

To a solution of thiazoline-thione **A3** (0.21g, 0.34 mmol, 1 equiv) was dissolved in 5 mL dichloromethane was added 2-trimethylsilyethanol (0.49 mL, 3.3 mmol, 10.0 equiv), followed by imidazole (35 mg, 0.51 mmol, 1.5 equiv). The resulting solution was stirred overnight, when TLC revealed complete disappearance of starting material **A3**. The reaction mixture was concentrated in vaccuo and submitted purified by flash-column chromatography (petroleum ether 40-60/ethyl acetate, 8:1) to provide compound **A4** (0.17 g, 93%) as a clear oil.

^{1}H NMR (400 MHz, $CDCl_3$) δ 7.46 – 7.40 (m, 6H), 7.32 – 7.27 (m, 6H), 7.26 – 7.19 (m, 3H), 5.59 (dtd, *J* = 14.4, 6.6, 1.2 Hz, 1H), 5.43 (ddt, *J* = 15.4, 6.2, 1.3 Hz, 1H), 4.51 – 4.39 (m, 1H), 4.27 – 4.12 (m, 2H), 2.90 (d, *J* = 4.0 Hz, 1H), 2.49 (d, *J* = 4.3 Hz, 1H), 2.22 (dd, *J* = 8.4, 7.1 Hz, 2H), 2.09 (q, *J* = 7.2 Hz, 2H), 1.07 – 0.94 (m, 2H), 0.05 (s, 9H); ^{13}C NMR (100 MHz, $CDCl_3$) δ 172.55, 144.87, 131.91, 130.16, 129.57, 127.85, 126.59, 68.58, 66.57, 63.08, 41.48, 31.43, 31.35, 17.31, -1.50.

The preparation was followed the reference and the characterization was in agreement with those reported[3].

*Synthesis of (S,E)-2-(trimethylsilyl)ethyl 3-(((S)-2-((((9H-fluoren-9- yl)methoxy)carbonyl)amino)-3-methylbutanoyl)oxy)-7-(tritylthio)hept-4-enoate (**AB1**)*

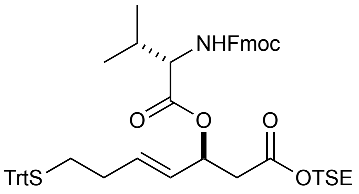

To a solution of compound **A4** (1.0 g, 1.93 mmol, 1 equiv) in dichloromethane (48 mL) at room temperature was added *N*-Fmoc-L-valine (3.3 g, 9.64 mmol, 5 equiv), EDCI–HCl (2.2 g, 11.6 mmol, 6 equiv), DMAP (23.6 mg, 0.19 mmol, 0.1 equiv), and DIPEA (2.0 mL, 11.6 mmol, 6 equiv). After stirring for 18 h, the reaction mixture was concentrated. The crude residue was purified by flash-column chromatography (petroleum ether 40-60/ethyl acetate, 20:1 to 5:1) to provide compound **AB1** (1.16 g, 72%) as a clear oil.

^{1}H NMR (400 MHz, $CDCl_3$) δ 7.77 (dq, *J* = 7.7, 1.1 Hz, 2H), 7.61 (dd, *J* = 7.5, 3.1 Hz, 2H), 7.45 – 7.15 (m, 19H), 5.81 – 5.52 (m, 2H), 5.48 – 5.26 (m, 2H), 4.53 – 4.32 (m, 2H), 4.31 – 4.15 (m, 4H), 2.68 (dd, *J* = 15.8, 7.9 Hz, 1H), 2.55 (dd, *J* = 15.9, 5.7 Hz, 1H), 2.20 (tdd, *J* = 9.8, 7.0, 1.7 Hz, 3H), 2.11 – 1.99 (m, 2H), 1.02 – 0.90 (m, 5H), 0.81 (d, *J* = 6.9 Hz, 3H), 0.04 (d, *J* = 9.4 Hz, 9H).

The preparation was followed the reference and the characterization was in agreement with those reported[6].

*Synthesis of (3S,4E)-2-(trimethylsilyl)ethyl-3-[(S)-2-((R)-2-{2-[(tert-butoxycarbonyl)methyl]thiazol-4-yl}-4-methyl-4,5-dihydrothiazole-4-carboxamido)-3-methylbutanoyloxy]-7-(tritylthio)hept-4-enoate (**ABC1**)*

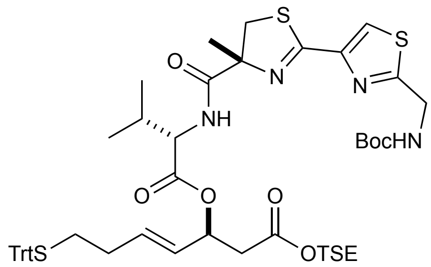

To a solution of Fmoc protected amine **AB1** (1.16 g, 1.38 mmol, 1 equiv) in acetonitrile (70 mL) at room temperature was added diethylamine (7.0 mL). After 2 h, the reaction mixture was concentrated under reduced vacuum, taken back up in ethyl acetate (35 mL), and concentrated again. In a separate flask, acid **C4** (542 mg, 1.52 mmol, 1.1 equiv), PyBOP (1.45 g, 2.76 mmol, 2

equiv), and DIPEA (0.72 mL, 4.14 mmol, 3 equiv) was combined in dichloromethane (25 mL). The freshly deprotected amine was added via acetonitrile (12.5 mL) to the flask containing the activated acid at room temperature. After 3 h, the resulting mixture was concentrated under reduced pressure. The crude residue was purified by flash-column chromatography (petroleum ether 40-60/ethyl acetate, 4:1 to 2:1) to provide amide **ABC1** (1.13 g, 86%) as a clear oil.

^{1}H NMR (500 MHz, $CDCl_3$) δ 7.90 (s, 1H), 7.40 – 7.36 (m, 6H), 7.30 – 7.15 (m, 9H), 5.74 – 5.56 (m, 2H), 5.37 (ddt, *J* = 15.4, 7.6, 1.4 Hz, 1H), 5.31 – 5.27 (m, 1H), 4.62 (d, *J* = 6.5 Hz, 2H), 4.48 (dd, *J* = 9.0, 4.7 Hz, 1H), 4.20 – 4.13 (m, 2H), 3.77 (d, *J* = 11.4 Hz, 1H), 3.32 (d, *J* = 11.5 Hz, 1H), 2.68 (dd, *J* = 15.7, 7.8 Hz, 1H), 2.55 (dd, *J* = 15.7, 5.7 Hz, 1H), 2.18 – 2.04 (m, 5H), 1.57 (s, 3H), 1.47 (s, 9H), 1.00 – 0.93 (m, 2H), 0.82 (d, *J* = 6.8 Hz, 3H), 0.74 (d, *J* = 6.9 Hz, 3H), 0.03 (s, 9H); ^{13}C NMR (125 MHz, $CDCl_3$) δ 174.43, 170.37, 169.66, 155.63, 148.66, 144.82, 133.91, 129.55, 128.03, 127.86, 127.77, 127.68, 126.60, 121.40, 121.35, 85.16, 80.46, 71.77, 66.59, 63.13, 56.79, 42.33, 41.50, 39.71, 31.30, 31.21, 31.16, 31.07, 28.33, 24.75, 19.05, 17.46, 17.28, -1.49.

The preparation was followed the reference and the characterization was in agreement with those reported[3, 6a].

*Synthesis of S-trityl macrocycle (**ABC3**)*

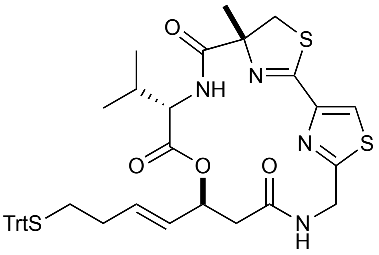

To a solution of linear precursor **ABC1** (0.10 g 0.104 mmol, 1 equiv) in dichloromethane (5 mL) at 0 °C was added TFA (1 mL). The reaction was allowed to warm to room temperature and stirred overnight. Solvents were evaporated, and the crude amino acid was taken up in toluene and concentrated a second time to remove residual TFA. The crude amino acid was then taken up in 5 mL dichloromethane and added dropwise to a stirred solution of DIPEA (0.122 mL, 0.702 mmol, 6 equiv) in dry acetonitrile (100 mL). The resulting moderately opaque solution was stirred for 10 min, before HATU (84.0 mg, 0.22 mmol, 2 equiv) and HOBt (30.0 mg, 0.22 mmol, 2 equiv) were added dropwise in 5 mL acetonitrile. The reaction was allowed to stir for 16 h, then concentrated

and submitted immediately to flash-column chromatography (petroleum ether 40-60/ethyl acetate, 9:1 to ethyl acetate) to afford the macrocycle **ABC3** (44.1 mg, 55%) as a clear oil.

^{1}H NMR (500 MHz, $CDCl_3$) δ 7.74 (s, 1H), 7.42 – 7.36 (m, 6H), 7.31 – 7.23 (m, 6H), 7.23 – 7.16 (m, 4H), 6.54 (dd, *J* = 9.2, 3.3 Hz, 1H), 5.72 (dtd, *J* = 15.1, 6.8, 1.1 Hz, 1H), 5.67 – 5.59 (m, 1H), 5.41 (ddt, *J* = 15.5, 6.5, 1.5 Hz, 1H), 5.20 (dd, *J* = 17.5, 9.2 Hz, 1H), 4.56 (dd, *J* = 9.4, 3.7 Hz, 1H), 4.12 (d, *J* = 14.3 Hz, 1H), 4.03 (d, *J* = 11.3 Hz, 1H), 3.27 (d, *J* = 11.3 Hz, 1H), 2.79 (dd, *J* = 16.2, 9.5 Hz, 1H), 2.66 (dd, *J* = 16.2, 3.2 Hz, 1H), 2.29 – 2.15 (m, 2H), 2.13 – 1.97 (m, 3H), 1.84 (s, 3H), 0.69 (d, *J* = 6.9 Hz, 3H), 0.53 (d, *J* = 6.8 Hz, 3H); ^{13}C NMR (125 MHz, $CDCl_3$) δ 173.49, 169.29, 168.73, 167.88, 164.43, 147.48, 144.76, 133.05, 129.55, 127.95, 127.88, 126.62, 124.03, 84.42, 71.76, 66.60, 57.84, 43.29, 40.99, 40.60, 34.00, 31.35, 31.21, 24.23, 18.84, 16.76.

The preparation was followed the reference and the characterization was in agreement with those reported[3].

Synthesis of ***Largazole Thiol***

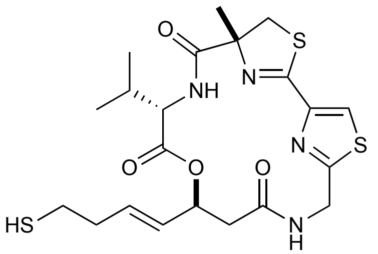

The *S*-trityl macrocycle **ABC3** (40.0 mg, 0.054 mmol, 1 equiv) was dissolved in dry dichloromethane (7 mL) and cooled to 0 °C. The mixture was successively treated with *i*Pr_3SiH (22.2 µL, 0.108 mmol, 2 equiv) and TFA (0.27 mL, to 0.2 M in **ABC3**). The reaction mixture was allowed to warm to room temperature and stirred for 1 h, before being concentrated and purified by flash-column chromatography (ethyl acetate) to provide **Largazole Thiol** (17.2 mg, 64%) as a clear oil.

^{1}H NMR (400 MHz, $CDCl_3$) δ 7.76 (s, 1H), 7.18 (d, *J* = 9.4 Hz, 1H), 6.50 (d, *J* = 8.8 Hz, 1H), 5.83 (dtd, *J* = 15.0, 6.9, 1.0 Hz, 1H), 5.72 – 5.64 (m, 1H), 5.54 (ddt, *J* = 15.5, 6.8, 1.4 Hz, 1H), 5.27 (dd, *J* = 17.6, 9.3 Hz, 1H), 4.60 (dd, *J* = 9.5, 3.5 Hz, 1H), 4.28 (dd, *J* = 17.6, 3.2 Hz, 1H), 4.04 (d, *J* = 11.3 Hz, 1H), 3.28 (d, *J* = 11.4 Hz, 1H), 2.86 (dd, *J* = 16.3, 10.0 Hz, 1H), 2.71 (dd, *J* = 16.3, 3.0 Hz, 1H), 2.61 – 2.51

(m, 2H), 2.41 – 2.31 (m, 2H), 2.15 – 2.05 (m, 1H), 1.86 (s, 3H), 0.70 (d, *J* = 6.9 Hz, 3H), 0.53 (d, *J* = 6.9 Hz, 3H); HRMS (ESI+) m/z: calcd. for $C_{21}H_{29}N_4O_4S_3$ $[M+H]^+$: 497.1351 found: 497.1331.

The preparation was followed the reference and the characterization was in agreement with those reported[3].

Synthesis of ***Largazole***

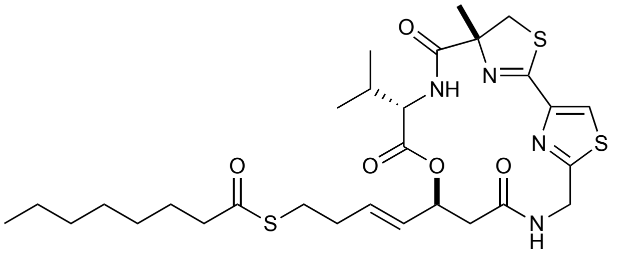

To a stirred solution of **Largazole Thiol** (14.9 mg, 0.03 mmol, 1 equiv) in dichloromethane (4 mL) at 0 °C were added triethylamine (8.4 µL, 0.06 mmol, 2 equiv) and octanoyl chloride (25.6 µL, 0.15 mmol, 5 equiv). After the mixture was stirred for 3 h at room temperature, the reaction was quenched with methanol at 0 °C. Then the mixture was concentrated in vacuo and purified by flash-column chromatography (ethyl acetate) to provide amide **Largazole** (8.5 mg, 45%) as a clear oil.

^{1}H NMR (400 MHz, $CDCl_3$) δ 7.76 (s, 1H), 7.16 (d, *J* = 9.3 Hz, 1H), 6.43 (d, *J* = 9.2 Hz, 1H), 5.83 (dt, *J* = 14.2, 6.8 Hz, 1H), 5.66 (t, *J* = 8.3 Hz, 1H), 5.52 (dd, *J* = 15.5, 6.9 Hz, 1H), 5.28 (dd, *J* = 17.6, 9.4 Hz, 1H), 4.61 (dd, *J* = 9.4, 3.4 Hz, 1H), 4.27 (dd, *J* = 17.6, 3.2 Hz, 1H), 4.05 (d, *J* = 11.3 Hz, 1H), 3.28 (d, *J* = 11.4 Hz, 1H), 2.90 (t, *J* = 7.2 Hz, 2H), 2.83 (d, *J* = 10.4 Hz, 1H), 2.69 (dd, *J* = 16.3, 2.9 Hz, 1H), 2.57 – 2.50 (m, 2H), 2.31 (q, *J* = 7.0 Hz, 2H), 2.11 (ddt, *J* = 10.3, 6.9, 3.4 Hz, 1H), 1.87 (s, 3H), 1.76 – 1.50 (m, 3H), 1.35 – 1.23 (m, 7H), 0.92 – 0.84 (m, 3H), 0.69 (d, *J* = 6.9 Hz, 3H), 0.52 (d, *J* = 6.8 Hz, 3H); HRMS (ESI+) m/z: calcd. for $C_{29}H_{43}N_4O_5S_3$ $[M+H]^+$: 623.2396 found: 623.2426.

The preparation was followed the reference and the characterization was in agreement with those reported[7].

*Synthesis of **Stapled Largazole***

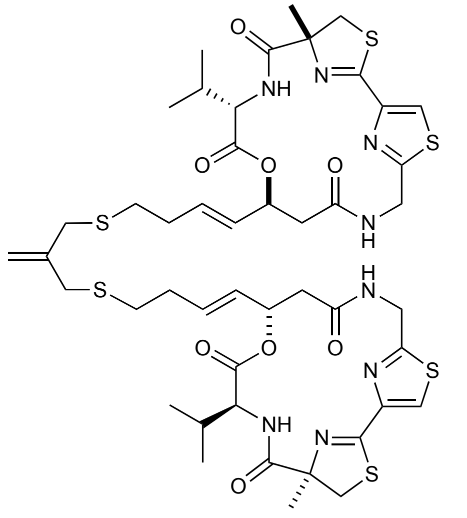

Largazole Thiol (9.0 mg, 0.018 mmol, 2 equiv) and potassium carbonate (6.3 mg, 0.045 mmol, 5 equiv) were dissolved in DMF (1 mL). 3-Bromo-2-bromomethyl-1-propene (0.52 µL, 0.005 mmol, 0.5 equiv) was added to the stirred solution. After being stirred at room temperature for 2 h, the same amount of 3-bromo-2-bromomethyl-1-propene was added and stirred for another 5 h. After this, the reaction was diluted with Et_2O (10 mL) and washed sequentially with a saturated solution of LiBr (10 mL) and brine (10 mL). The organic layer was dried with magnesium sulphate, filtered, and concentrated under reduced pressure. The resulting residue was purified by flash-column chromatography (ethyl acetate) to provide the **Stapled Largazole** (6.5 mg, 69%) as white solid.

^{1}H NMR (500 MHz, $CDCl_3$) δ 7.76 (s, 2H), 7.18 (d, *J* = 9.5 Hz, 2H), 6.57 (dd, *J* = 9.4, 3.3 Hz, 2H), 5.87 (dd, *J* = 14.6, 7.7 Hz, 2H), 5.73 – 5.63 (m, 2H), 5.27 (dd, *J* = 17.6, 9.3 Hz, 2H), 4.97 (s, 2H), 4.60 (dd, *J* = 9.4, 3.5 Hz, 2H), 4.25 (dd, *J* = 17.6, 3.3 Hz, 2H), 4.03 (d, *J* = 11.4 Hz, 2H), 3.32 – 3.15 (m, 6H), 2.86 (dd, *J* = 16.3, 10.0 Hz, 2H), 2.71 (dd, *J* = 16.3, 3.0 Hz, 2H), 2.45 (t, *J* = 7.2 Hz, 4H), 2.31 (q, *J* = 6.9 Hz, 4H), 2.10 (pd, *J* = 6.9, 3.6 Hz, 2H), 1.86 (s, 6H), 0.69 (d, *J* = 6.9 Hz, 6H), 0.52 (d, *J* = 6.8 Hz, 6H); ^{13}C NMR (125 MHz, $CDCl_3$) δ 173.71, 169.50, 169.03, 168.10, 164.69, 162.67, 147.63, 140.88, 133.39, 128.08, 124.27, 115.74, 84.58, 72.14, 57.94, 43.48, 41.26, 40.67, 36.63, 35.56, 34.29, 32.06, 31.59, 30.50, 29.85, 24.40, 19.06, 16.83; HRMS (ESI+) m/z: calcd. for $C_{46}H_{61}N_8O_8S_6$ $[M+H]^+$: 1045.2931 found: 1045.2901.

Table S2. The gradient of eluent for Stapled Largazole thiol.

Solvents	0 min	30 min	35 min	40 min	41 min
B	80%	10%	10%	80%	80%
C	20%	90%	90%	20%	20%

Flow rate: 3.5 mL/min

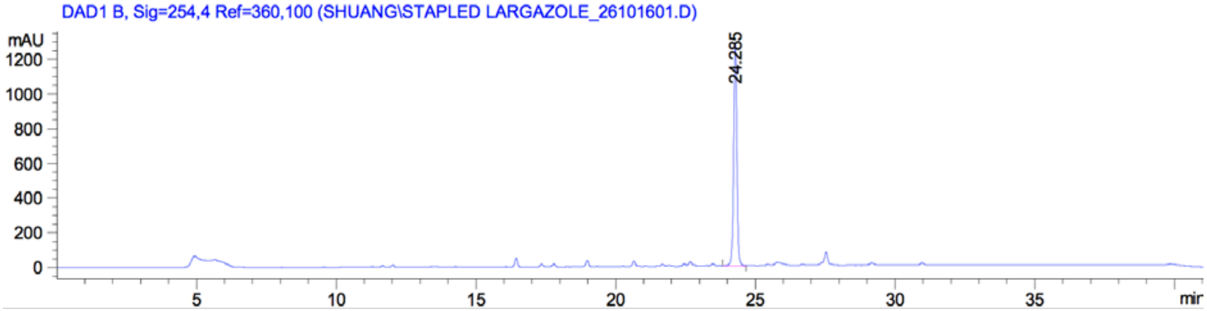

Figure S4. HPLC chromatogram of Stapled Largazole thiol.

Synthesis of ***Unstapled Largazole thiol***

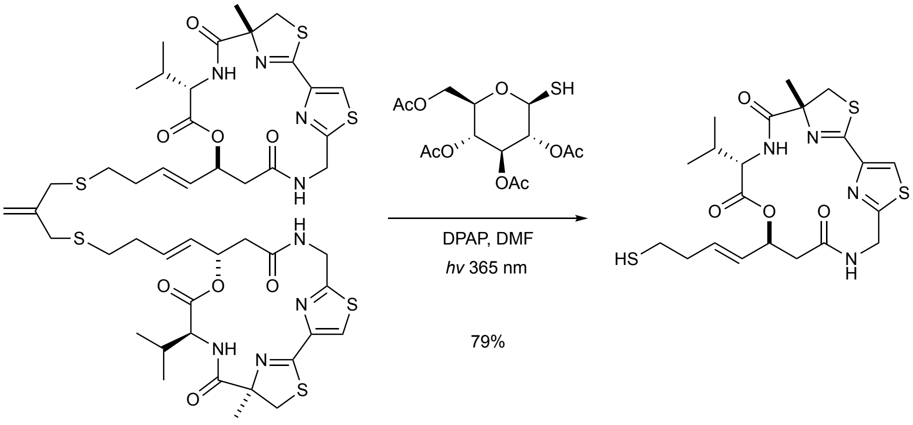

Scheme S5. Unstapling of stapled Largazole.

The **Stapled Largazole thiol** (4.0 mg, 3.8 µmol, 1 equiv) was dissolved in DMF (1.5 mL) at room temperature. The DPAP (7 µL, 0.05 M in DMF, 0.38 µmol, 0.1 equiv) and 4AcGlcSH (120 µL, 0.1 M in DMF, 11.5 µmol, 3 equiv) was added to the solution. The mixture was degassed with argon, followed by irradiated with UV light for 30 min. After being stirred in air for 10 min, TCEP (5.2 mg, 20.9 µmol, 6 equiv) was added to the solution. After 1 h, the solvent was removed under reduced pressure and purified by HPLC to afford the **Largazole thiol** (3.0 mg, 79%) as a white solid. HRMS (ESI+) m/z: calcd. for $C_{21}H_{29}N_4O_4S_3$ $[M+H]^+$: 497.1351 found: 497.1331.

The gradient of eluent for **Unstapled Largazole thiol** is the same as **Stapled Largazole thiol**.

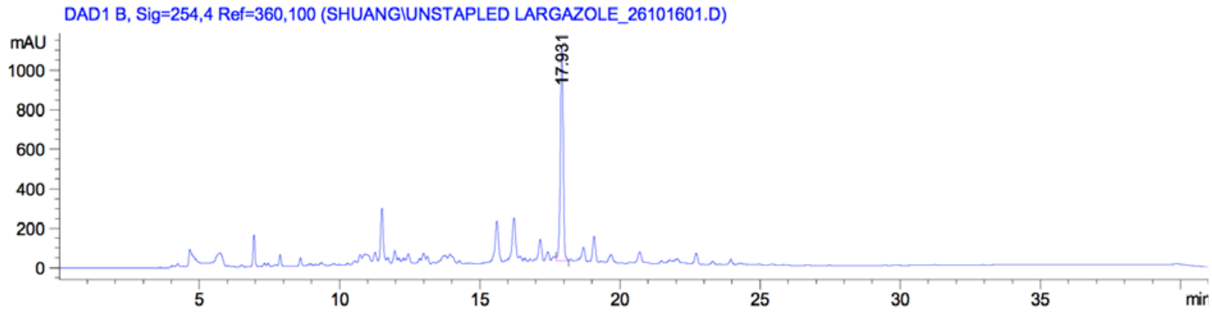

Figure S5. HPLC chromatogram of unstapling reaction.

Computational Details

Full geometry optimizations and transition structure (TS) searches were carried out with Gaussian 16[8] using the M06-2X hybrid functional[9] and 6-311++G(2d,p) basis set with ultrafine integration grids. Bulk solvent effects in water were considered implicitly through the IEF-PCM polarizable continuum model.[10] Radical species were calculated as open-shell doublets. The possibility of different conformations was taken into account for all structures. All stationary points were characterized by a frequency analysis performed at the same level used in the geometry optimizations from which thermal corrections were obtained at 298.15 K. The quasiharmonic approximation reported by Truhlar *et al.* was used to replace the harmonic oscillator approximation for the calculation of the vibrational contribution to enthalpy and entropy.[11] Scaled frequencies were not considered. Mass-weighted intrinsic reaction coordinate (IRC) calculations were carried out by using the Gonzalez and Schlegel scheme[12] in order to ensure that the TSs indeed connected the appropriate reactants and products. Gibbs free energies (ΔG) were used for the discussion on the relative stabilities of the considered structures. Free energies calculated using the gas phase standard state concentration (1 atm = 1/24.5 M) were converted to reproduce the standard state concentration in solution (1 M) by adding or subtracting 1.89 kcal mol^{-1} for bimolecular additions and decompositions, respectively. The lowest energy conformer for each calculated stationary point (Supplementary Figure S6) was considered in the discussion; all the computed structures can be obtained from authors upon request. Cartesian coordinates, electronic energies, entropies, enthalpies, Gibbs free energies, and lowest frequencies of the calculated structures are summarized in Supplementary Table S3.

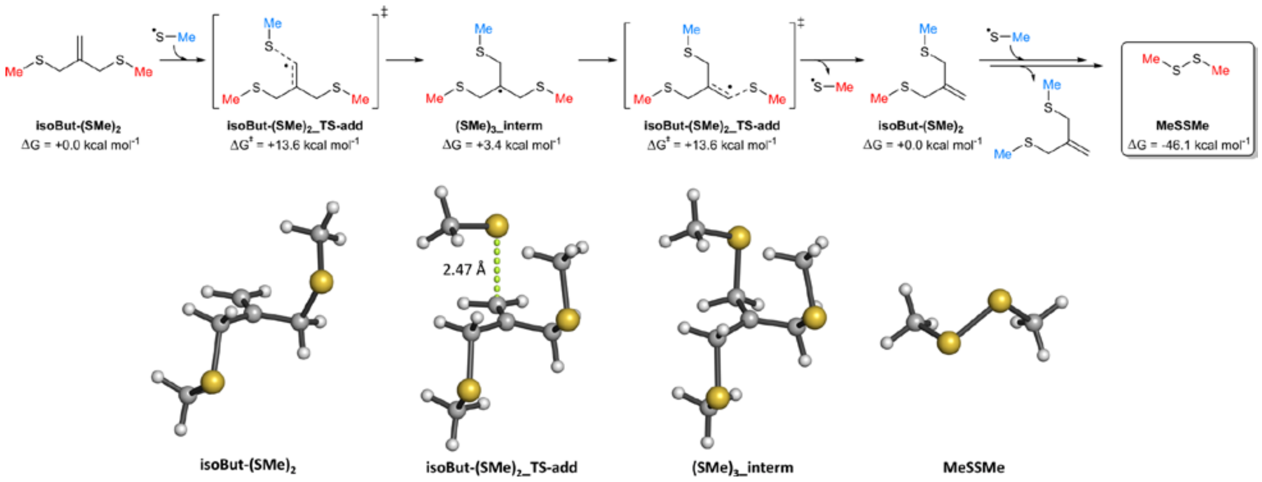

Figure S6. Guide to the calculated mechanism, labeling of calculated structures and relative free energies (only the lowest energy conformers are shown)

Table S3. Energies, entropies, and lowest frequenciesof the lowest energy calculated structures.[a]

Structure	E_{elec} (Hartree)	E_{elec} + ZPE (Hartree)	H (Hartree)	S (cal mol^{-1} K^{-1})	G (Hartree)	Lowest freq. (cm^{-1})	# of imag freq.
**SMe**	–438.040869	–438.005209	–438.001045	59.9	–438.029492	532.4	0
**isoBut-(SMe)$_2$**	–1032.173871	–1032.005790	–1031.993779	105.6	–1032.042103	34.9	0
**isoBut-(SMe)$_2$_TS-add**	–1470.217078	–1470.010970	–1469.995257	126.3	–1470.052981	–209.7	1
**(SMe)$_3$_interm**	–1470.235089	–1470.027810	–1470.012032	127.3	–1470.069203	33.0	0
**MeSSMe**	–876.183104	–876.105223	–876.097853	79.5	–876.135529	89.3	0

[a]Energy values calculated at the PCM(H_2O)/M06-2X/6-311++G(2d,p) level. 1 Hartree = 627.51 kcal mol^{-1}. Thermal corrections at 298.15 K.

Cartesian coordinates of the lowest energy structures calculated with PCM(H_2O)/M06-2X/6-311++G(2d,p)

Structure **SMe**

```
S   -0.690199   0.000018  -0.001904
C    1.105956   0.000107  -0.008003
H    1.414476  -0.006139   1.041076
H    1.496285  -0.894929  -0.486255
H    1.496692   0.900142  -0.476333
```

Structure **isoBut-(SMe)2**

```
S   -2.283852  -0.928009   0.097972
C   -3.210952   0.556571  -0.363966
C   -0.863325  -0.145811   0.941656
H   -4.114204   0.220837  -0.869398
H   -3.482408   1.121985   0.526541
H   -2.626364   1.178564  -1.038864
H   -0.306166  -0.975124   1.380201
H   -1.247003   0.482006   1.745460
C   -0.000001   0.638867   0.000001
C    0.863323  -0.145809  -0.941655
C    0.000000   1.969233   0.000002
H    0.306165  -0.975123  -1.380201
H    1.247002   0.482008  -1.745458
H    0.614989   2.531025  -0.693954
H   -0.614988   2.531025   0.693960
S    2.283851  -0.928009  -0.097971
C    3.210955   0.556569   0.363962
H    4.114208   0.220834   0.869392
H    3.482410   1.121983  -0.526546
H    2.626370   1.178565   1.038861
```

Structure **isoBut-(SMe)2_TS-add**

```
S   -2.672563  -0.337523  -0.704444
C   -3.068184  -1.620365   0.511703
C   -0.855618  -0.541301  -0.772210
H   -4.152729  -1.648542   0.595068
H   -2.705901  -2.588856   0.169968
H   -2.637744  -1.379982   1.482439
H   -0.534839   0.173871  -1.532557
H   -0.630415  -1.551123  -1.113626
C   -0.209561  -0.262149   0.543976
C   -0.360462   1.101391   1.154541
C    0.449219  -1.240985   1.215007
H   -1.373398   1.206825   1.552771
H    0.341531   1.225448   1.979489
H    0.795674  -1.074335   2.227452
H    0.394861  -2.268505   0.876089
S   -0.195464   2.507251   0.010767
C    1.533270   2.306690  -0.486423
H    1.764183   3.131027  -1.158404
H    2.185482   2.349026   0.383923
H    1.679878   1.361260  -1.006148
S    2.779670  -1.090867   0.397550
C    2.390343  -1.593626  -1.296055
H    1.837855  -0.819480  -1.826714
H    3.342488  -1.747786  -1.805837
H    1.832733  -2.528563  -1.309104
```

Structure **(SMe3)_interm**

```
S    2.774428  -0.132031   0.601601
C    2.991980  -1.489434  -0.578308
C    0.964645  -0.312632   0.927421
H    4.050318  -1.527846  -0.827953
H    2.693275  -2.435120  -0.127938
H    2.416532  -1.307569  -1.485330
H    0.753871   0.501328   1.625413
H    0.809550  -1.264722   1.435056
C    0.148998  -0.215293  -0.303387
C    0.141753   1.039836  -1.104742
C   -0.539915  -1.407627  -0.849372
H    1.112726   1.196534  -1.588829
H   -0.618128   0.990535  -1.886358
H   -0.488299  -1.445731  -1.939576
H   -0.130089  -2.335757  -0.449016
S   -0.113941   2.577638  -0.150154
C   -1.788069   2.273614   0.468402
H   -2.079216   3.151894   1.041184
H   -2.477552   2.133172  -0.362124
H   -1.805918   1.397889   1.115268
S   -2.353786  -1.417050  -0.495377
C   -2.293609  -1.634198   1.301512
H   -1.779996  -0.800636   1.778435
H   -3.322680  -1.666137   1.653615
H   -1.796315  -2.570344   1.551876
```

Structure **MeSSMe**

```
S    0.911181  -0.507200   0.481035
S   -0.911180  -0.507197  -0.481037
C    1.803835   0.817506  -0.384582
H    1.300572   1.770699  -0.242677
H    2.794128   0.859545   0.068689
H    1.891963   0.579918  -1.441312
C   -1.803835   0.817501   0.384585
H   -1.300574   1.770697   0.242690
H   -2.794126   0.859546  -0.068691
H   -1.891971   0.579910   1.441313
```

Biological Assays

Parallel Artificial Membrane Permeability Assay (PAMPA)

The PAMPA Evolution^{TM} instrument was used to determine permeability. In PAMPA, a sandwich is formed such that each composite well is divided into two chambers, separated by a 125 µm thick microfilter disc (0.45 µm pores), coated with Pion GIT-0 phospholipid mixture. The effective permeability, P_e, of each compound was measured at the customer-specified pHs in the donor compartment using low-binding, low UV Prisma buffer. The drug-free acceptor compartment was filled with acceptor sink buffer containing a scavenger at the start of the test. The proprietary scavenger mimics serum proteins and blood circulation, thus creating sink conditions.

In the default protocol the aqueous solutions of studied compounds are prepared by diluting and thoroughly mixing 3 µL of DMSO stock in 600 µL of Prisma HT buffer. Final concentration of organic solvent (DMSO) in aqueous buffer is ≤ 0.5% (v/v).

The reference solution is identical to the donor at time zero, so that any surface adsorption effects from the plastic is compensated. The PAMPA sandwich was assembled and allowed to incubate for ~15 hours. The solutions in the donor compartment were un-stirred within duration of the experiment. Thus, the thickness of the aqueous boundary layer expected to be about 1000 µm. The sandwich was then separated, and both the donor and receiver compartments were assayed for the amount of drug present by comparison with the UV spectrum obtained from reference standards. Mass balance was used to determine the amount of material remaining in the membrane filter and on the plastic (%R). Ketoprofen, Verapmil and Propanolol were used as reference compounds.

Buffers preparation

pH of Prisma HT buffer was adjusted to the requested values using 1.0 M solution of NaOH. Actual pH the buffers was 7.40 ± 0.05.

Stock solutions preparation

Sample powders pre-weighed in glass vials were brought to the room temperature at the day of the experiment. The samples were diluted with an organic solvent (DMSO) to prepare stock solutions at concentration ~10 mM. The stock solutions were further diluted in buffer at 7.40

producing the aqueous sample solutions at concentrations ~50 µM. The amount of DMSO in the resulting solution was <0.5% (v/v). The solutions were filtered prior assaying the samples.

Table S4. The parallel artificial membrane permeability assay (PAMPA).

Compound	pH	Avg. P_e	SD P_e	Avg. %R	SD %R	Avg. logP_e	SD logP_e	Domin, nm
Stapled Largazole	7.4	5.3	1.7	10	1	-5.29	0.14	240-498

P_e - effective permeability (x10^{-6} cm/sec) measured directly from assay
pH - refers to the values in donor compartment. Acceptor had a special sink buffer (ASB) at pH 7.4.
%R – membrane retention.
Avg – the value is reported as an average of quadruplicates

Cell Culture

HCT-116 (ATCC® CCL-247™), kindly provided by Dr Marco Di Antonio (Department of Chemistry, University of Cambridge, UK), was maintained at 37 °C humidified air, 5% CO_2, and assayed in McCoy's 5A (Modified) Medium (Gibco™, Thermo Fisher Scientific) supplemented with 10% FBS (Gibco™, Thermo Fisher Scientific), 100 units/mL penicillin and 100 µg/mL streptomycin (Gibco™, Thermo Fisher Scientific). The cells were split before reaching confluence with 0.05% Trypsin-EDTA (Gibco™, Thermo Fisher Scientific).

Cell viability assay

HCT-116 cells suspended in McCoy's 5A medium were plated in 96-well plates (200 µL, 2,500 cells/well), incubated (37 °C, 5% CO_2), and 24 h later treated with various concentrations of compounds or solvent control (0.25% DMSO). After another 48 h of incubation, by adding 20 µL CellTiter-Blue® solution to each well and incubating 6 h. Fluorescence was then measured (Ex/Em = 555 nm / 585 nm), using the the SpectraMax® i3x Multi-Mode Microplate Reader. Cell viability was calculated according to the manufacturer's instructions (Promega).

Table S5. GI_{50} of the Largazole, Largazole Thiol and Stapled Largazole

Compound	GI_{50}/nM
Largazole	13.56
Largazole Thiol	191.7
Stapled Largazole	529.9

UV Condition Screening

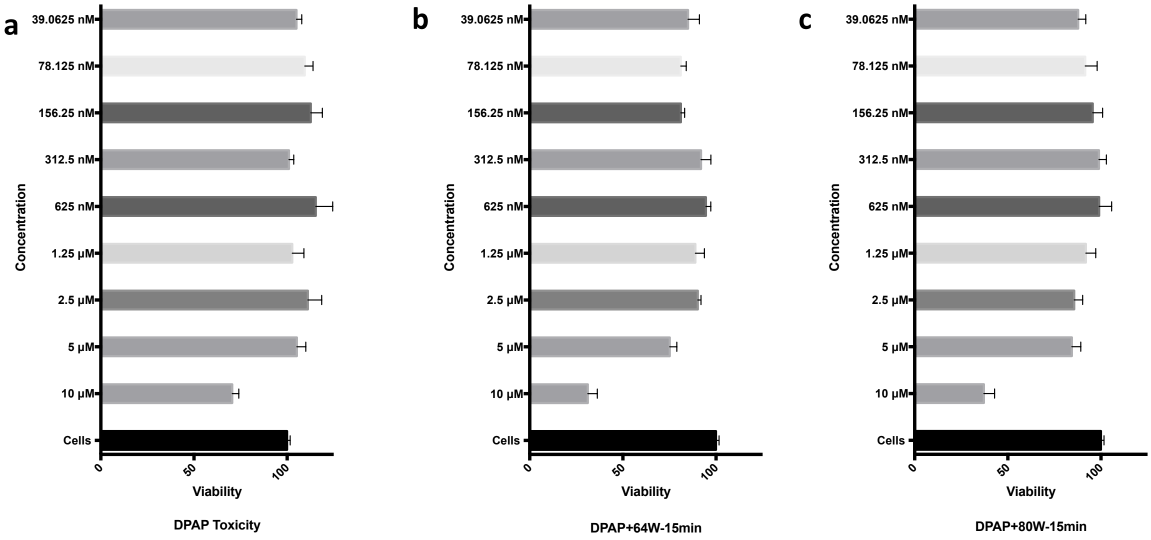

Figure S7. The DPAP concentration and UV condition Screening. a) The cytotoxicity of DPAP without UV; b) The cytotoxicity of DPAP with UV, 365 nm, 64W, 15 min; c) The cytotoxicity of DPAP with UV, 365 nm, 80W, 15 min.

Photoactivation of Stapled Largazole in HCT-116 cells

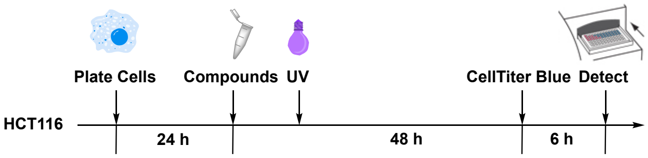

Scheme S6. The photoactivation of Stapled Largazole in HCT-116 cells.

HCT-116 cells were plated in 200 µL of medium per well at a density of 2,500 cells/well and grown for 24 h in two sterile 96-well solid bottom plates. incubated (37 °C, 5% CO_2). After 24 h, the cells were treated with various compounds (Largazole, Largazole Thiol and Stapled Largazole at 150 nM, DPAP at 1.5 µM) or solvent control (0.25% DMSO). Then the UV group plate were irradiated with UVA light (365 nm, 80 W) for 15 min. After another 48 h of incubation, the cell viability was measured according to the general method above.

Histone Deacetylase (HDAC) Activity Assay (Fluorometric)

Following the measurement of the cell viability, the plates were washed with cold PBS for three times. Then, the whole-cell lysates were prepared on ice using RIPA Lysis and Extraction Buffer

(Thermo Scientific™). The activity assay was carried out with the cell lysates following the manufacturer's instruction (Abcam). Briefly, 20 µL ddH_2O, 5 µL HDAC Assay Buffer and 5 µL Substrate Peptide were added to 96-well plate. Then the reactions were initiated by adding 20 µL cell lysates or buffer for no enzyme control to each well and mixed thoroughly at RT. After being incubated for 20 min, 20 µL of Stop Solution was added to each well and mixed thoroughly, followed by 5 µL of Developer solution. After being incubated for another 30 min at RT, the fluorescence intensity was read at Ex/Em = 355 nm / 460 nm.

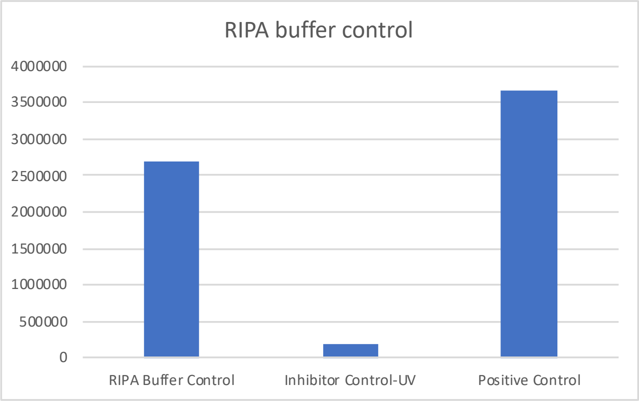

Figure S8. RIPA buffer control with HDAC activity kit.

NMR Spectrum

^{1}H NMR of **C1**

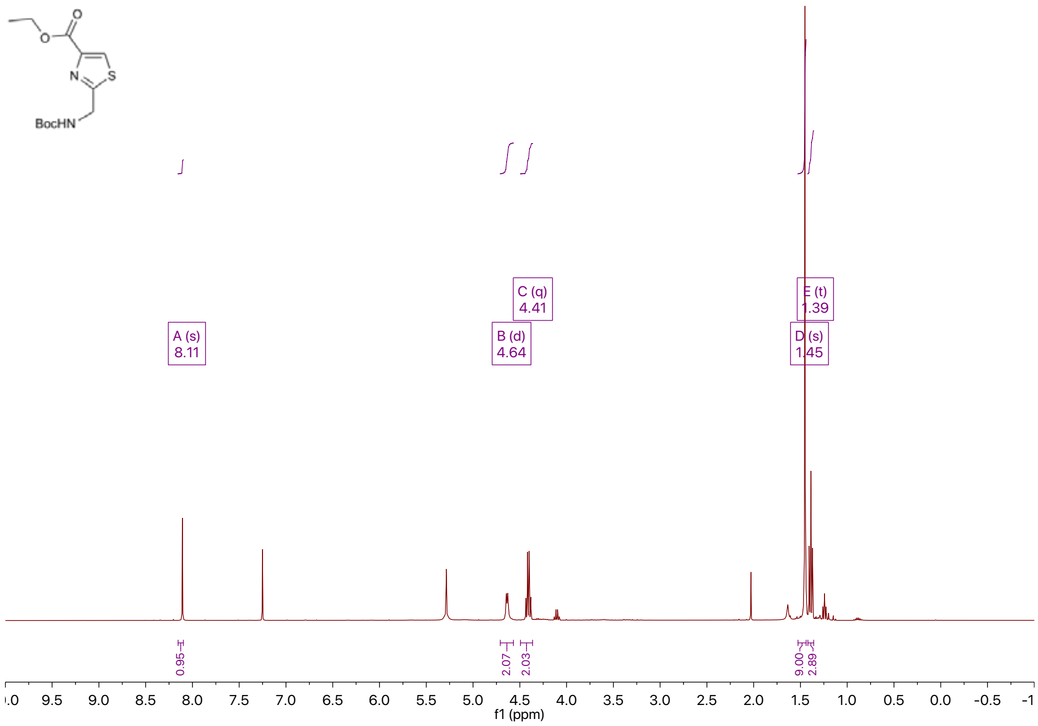

^{13}C NMR of **C1**

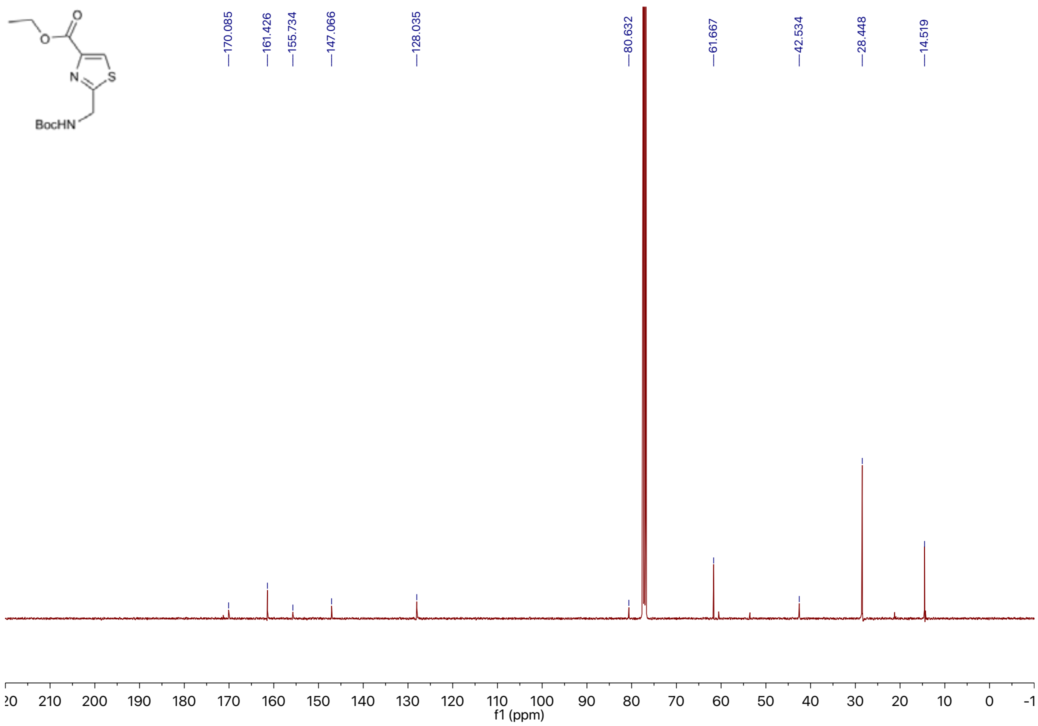

^{1}H NMR of **C2**

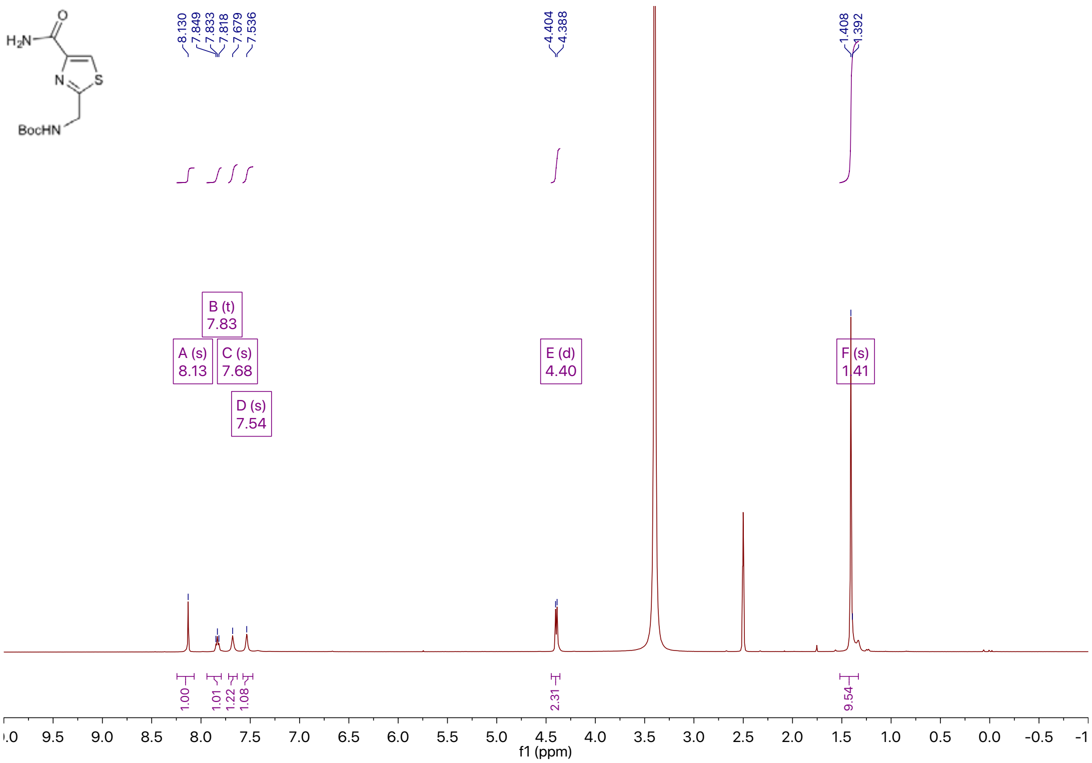

^{13}C NMR of **C2**

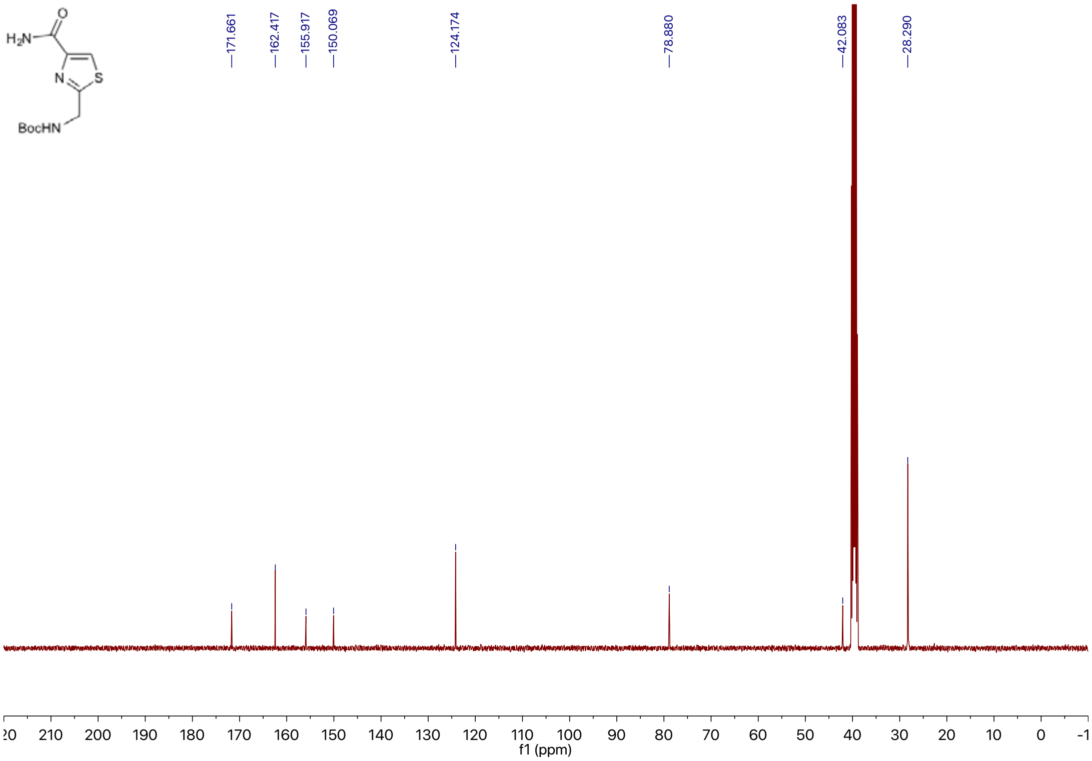

^{1}H NMR of **C3**

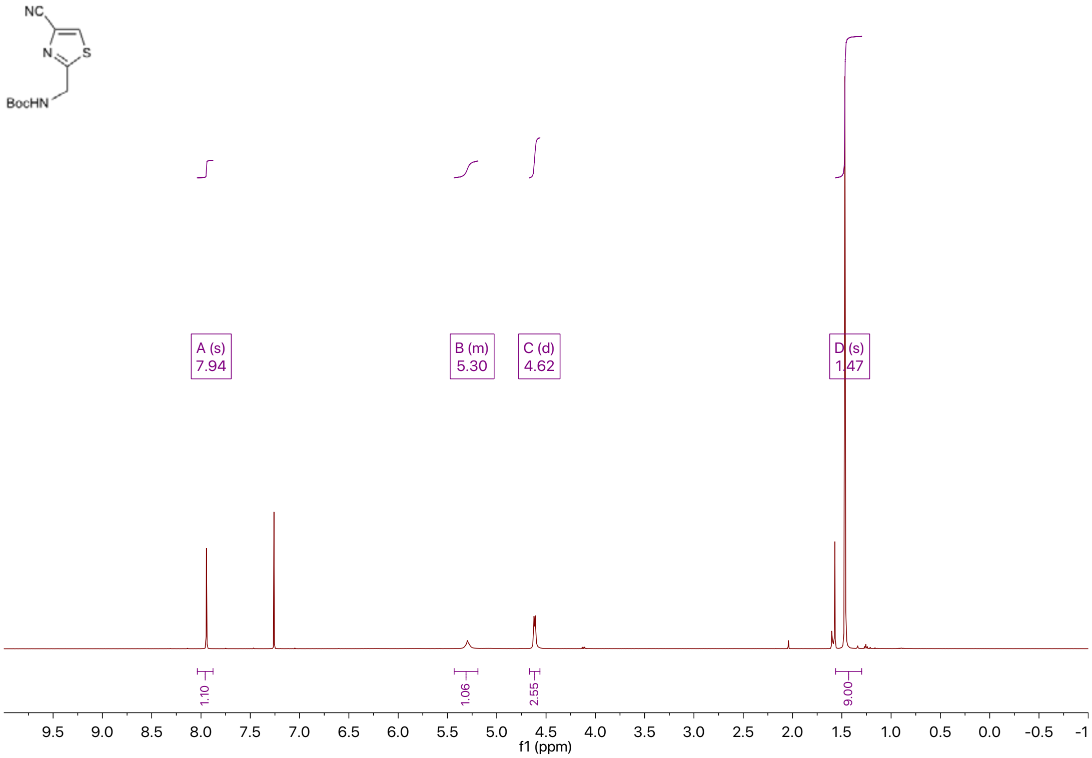

^{13}C NMR of **C3**

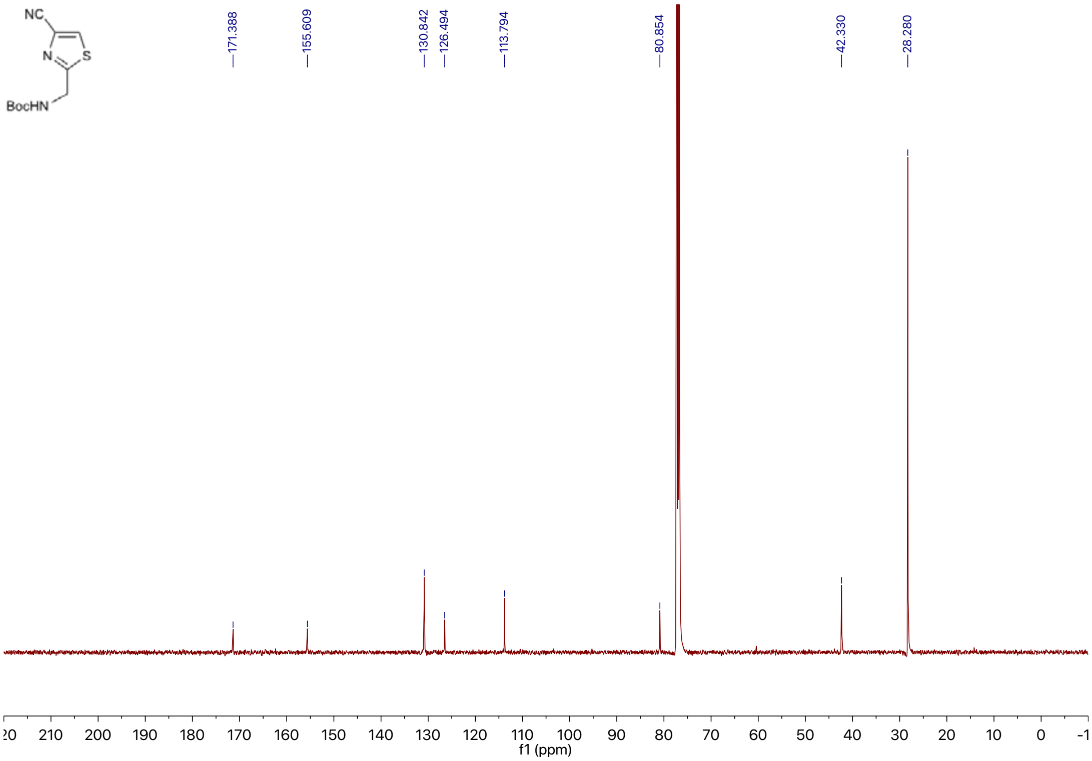

^{1}H NMR of **C4**

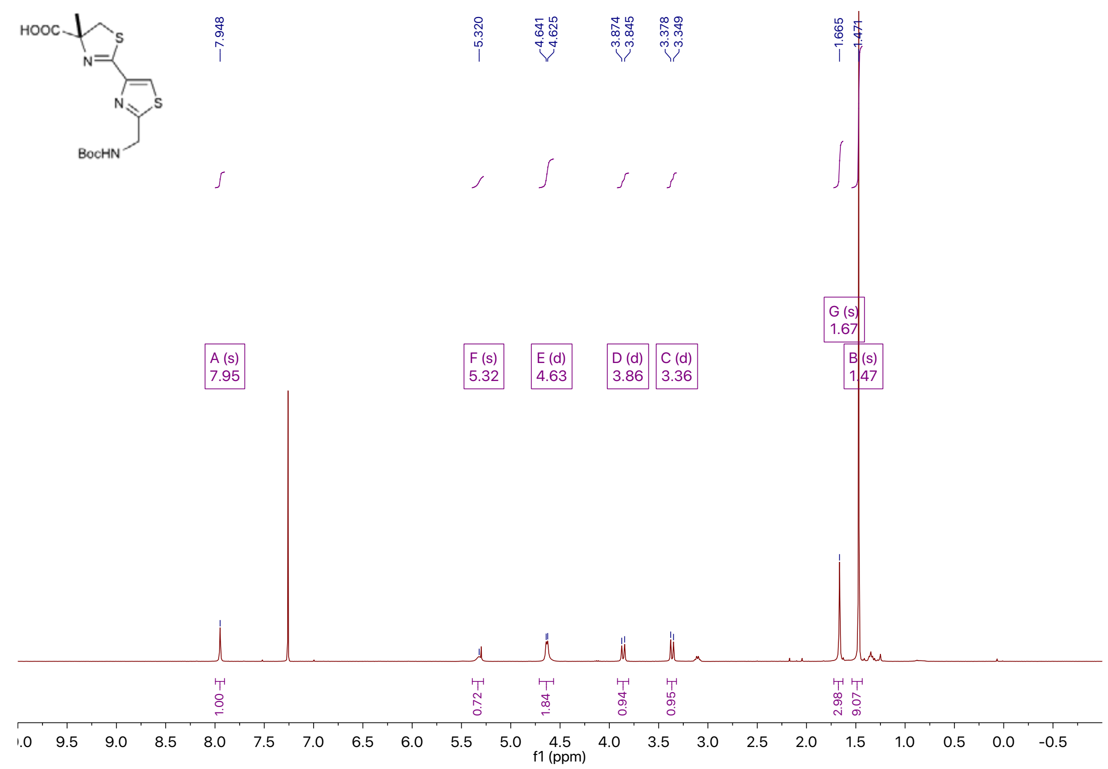

^{13}C NMR of **C4**

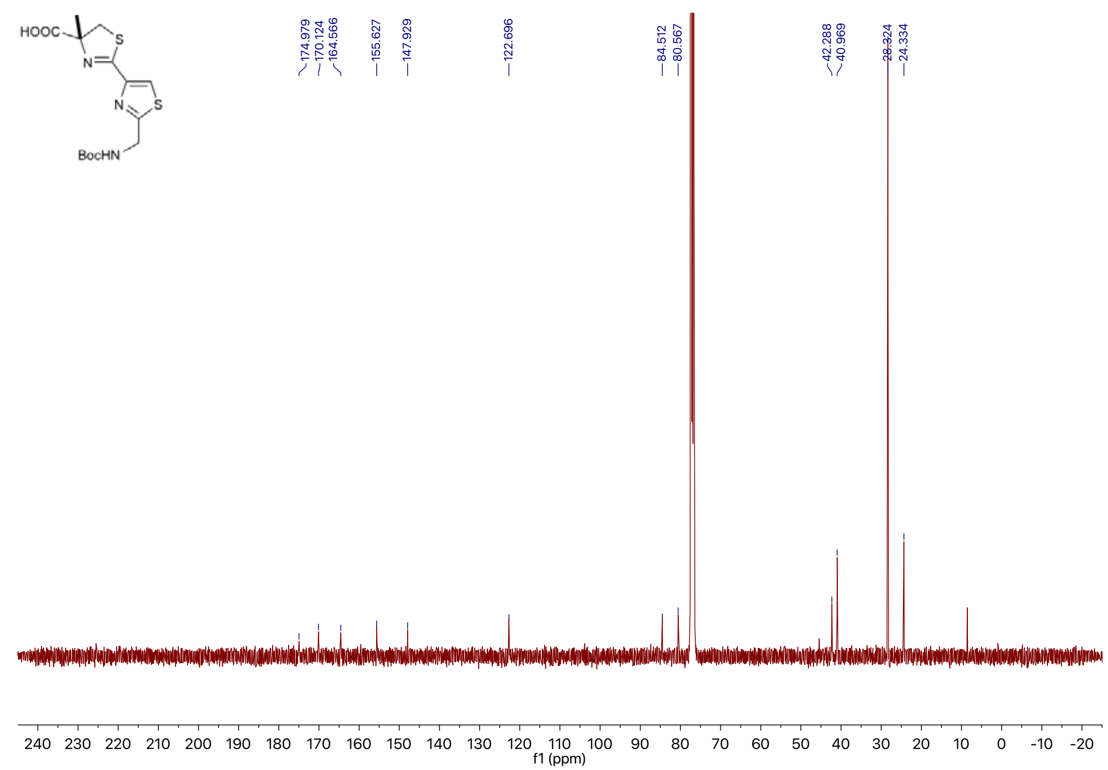

^{1}H NMR of **Auxiliary**

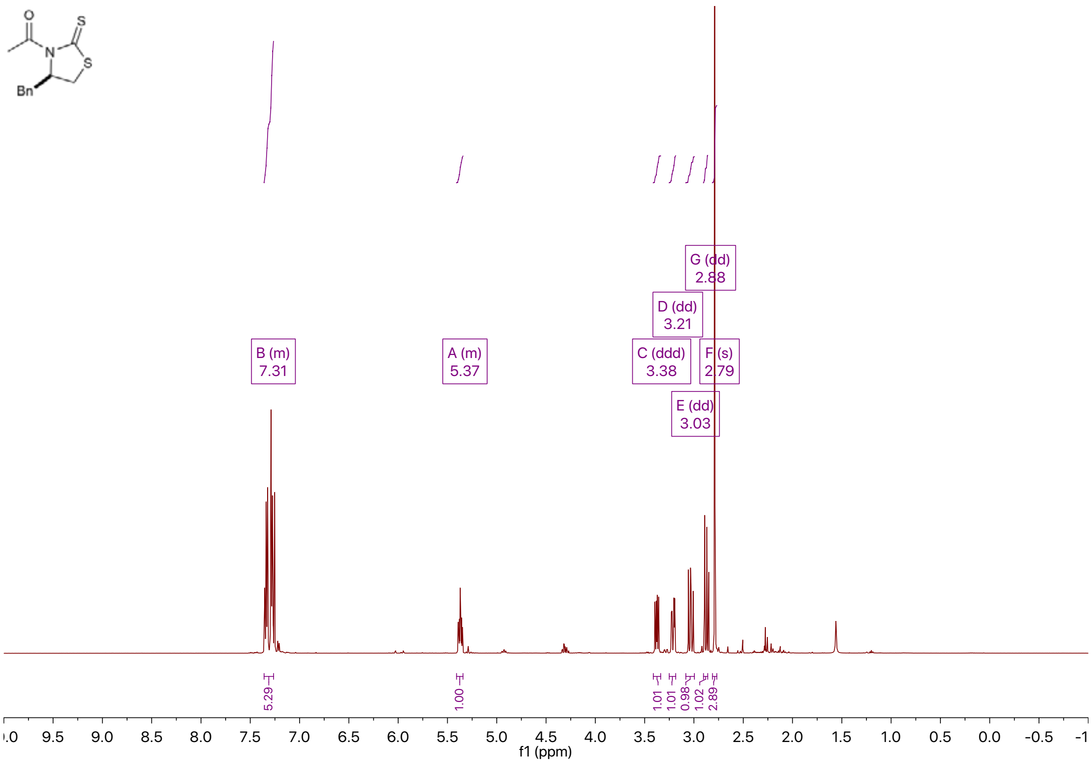

^{13}C NMR of **Auxiliary**

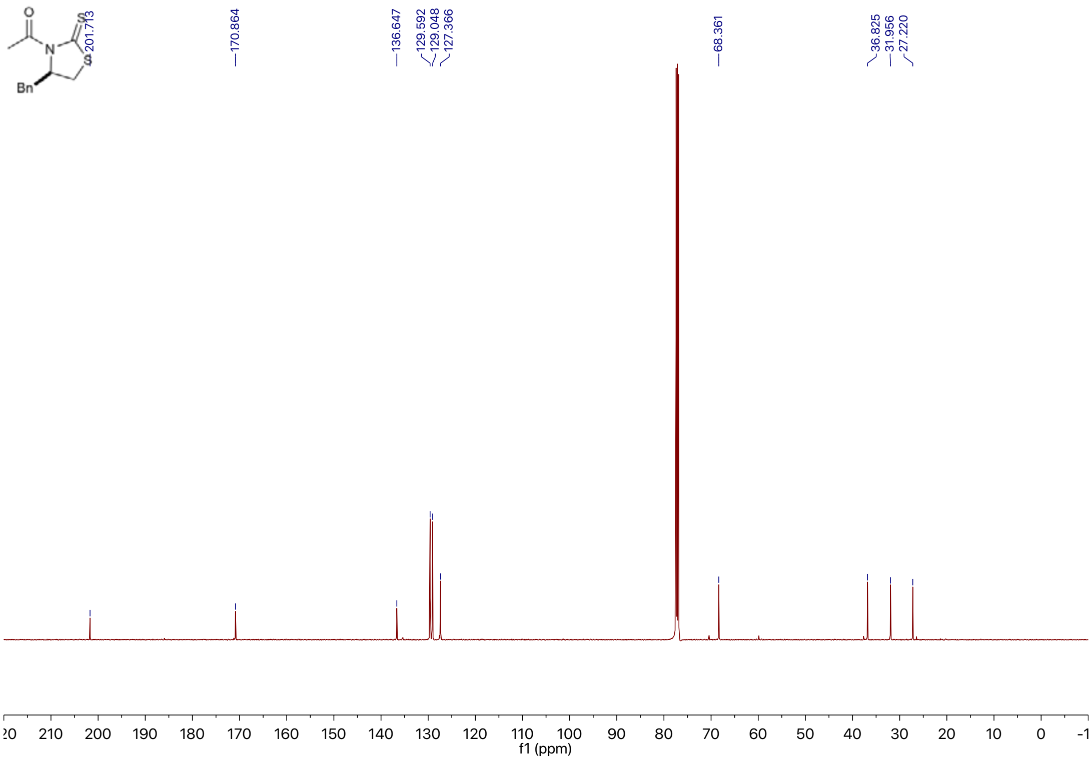

^{1}H NMR of **A2**

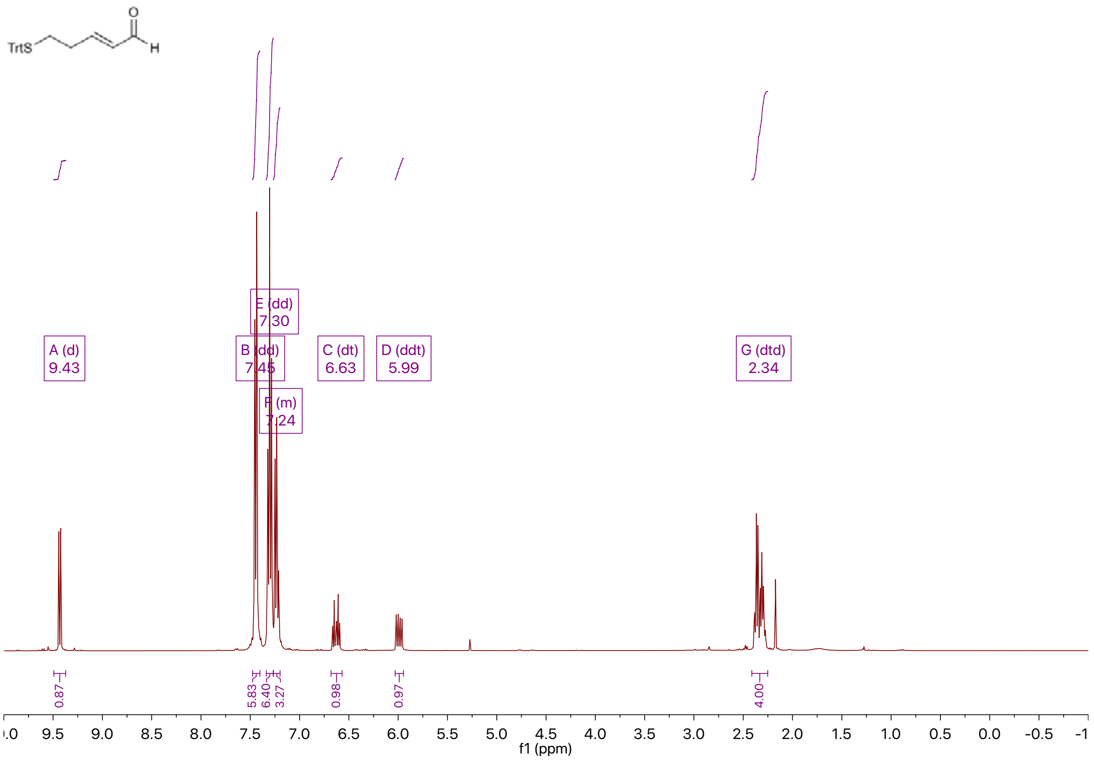

^{13}C NMR of **A2**

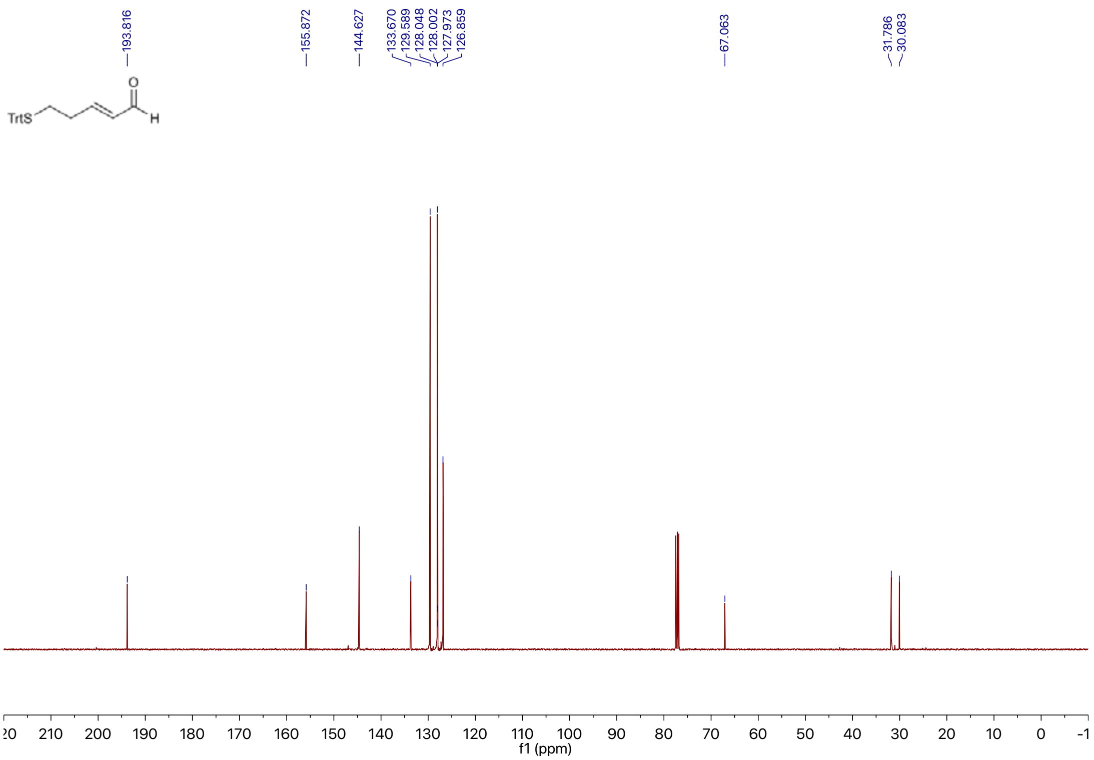

^{1}H NMR of **A3**

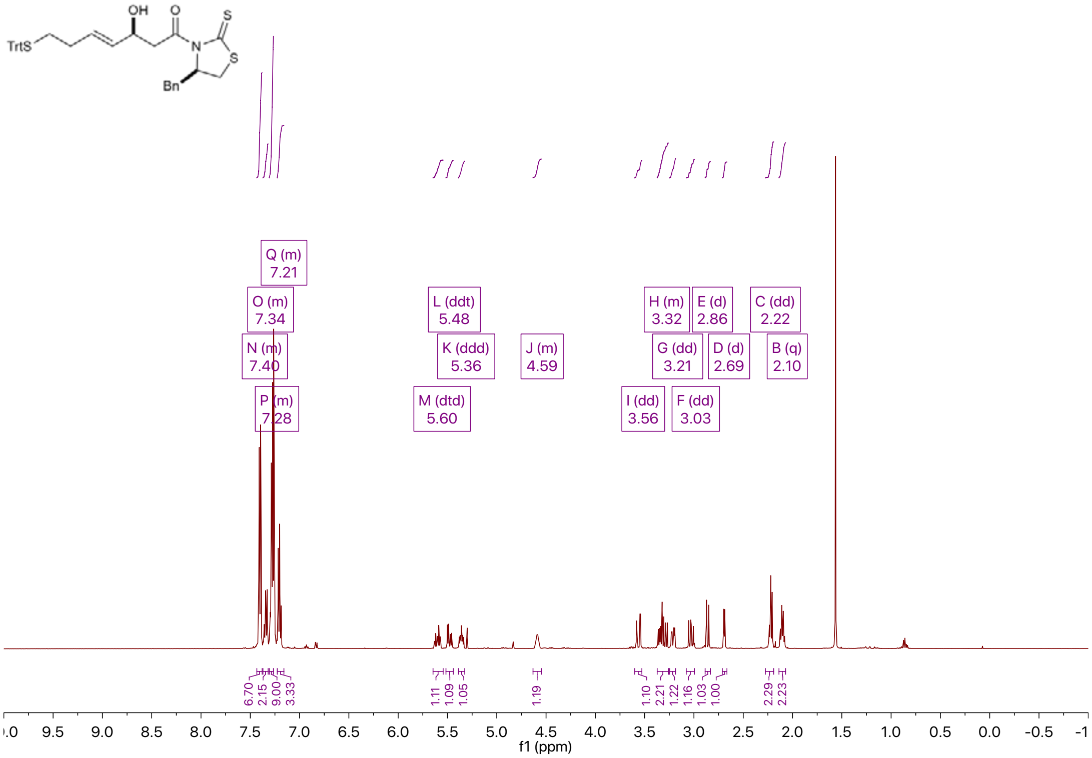

^{13}C NMR of **A3**

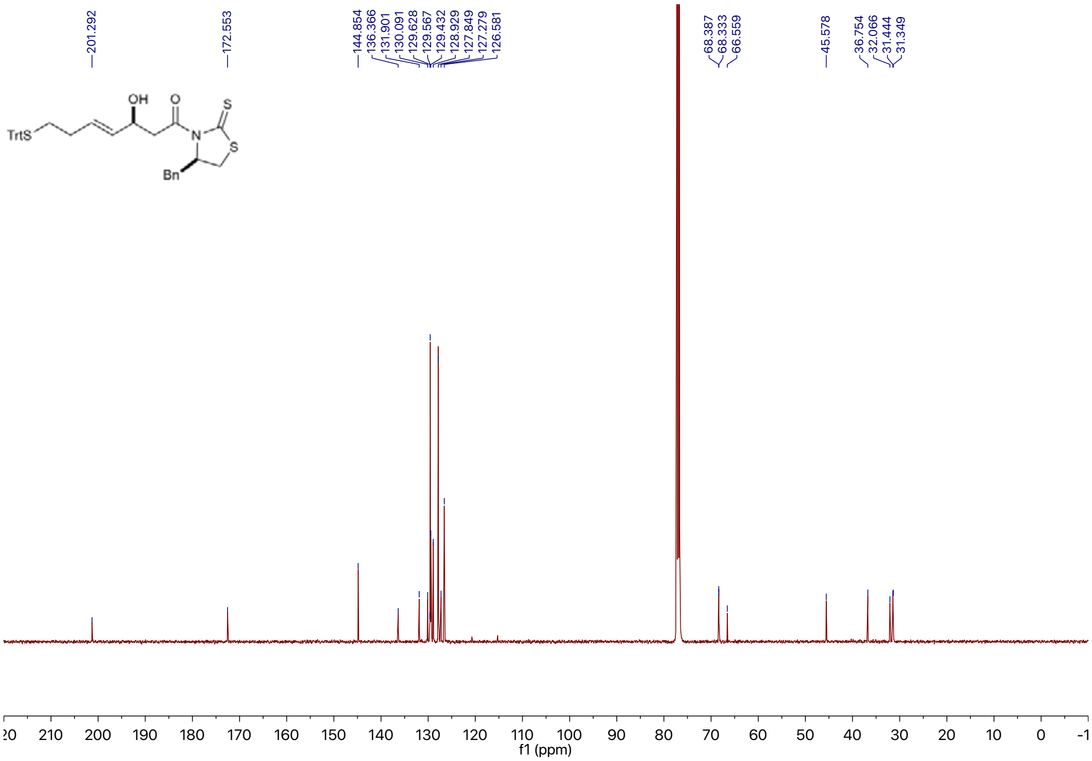

^{1}H NMR of **A4**

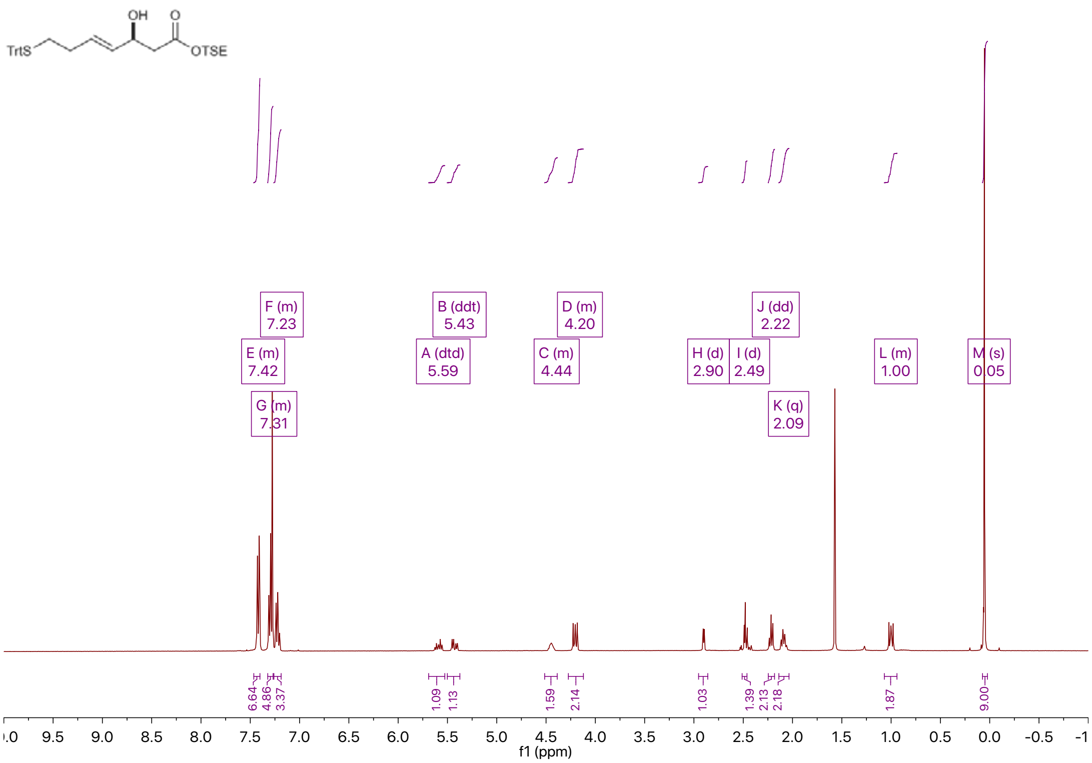

^{13}C NMR of **A4**

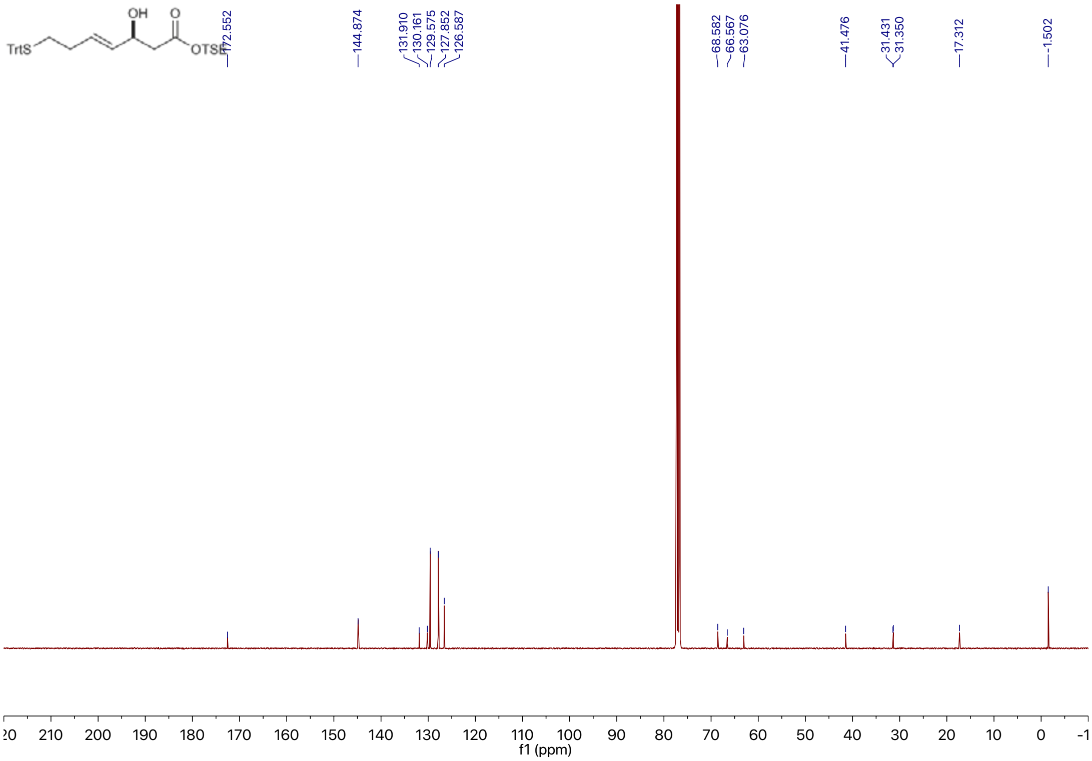

^{1}H NMR of **AB1**

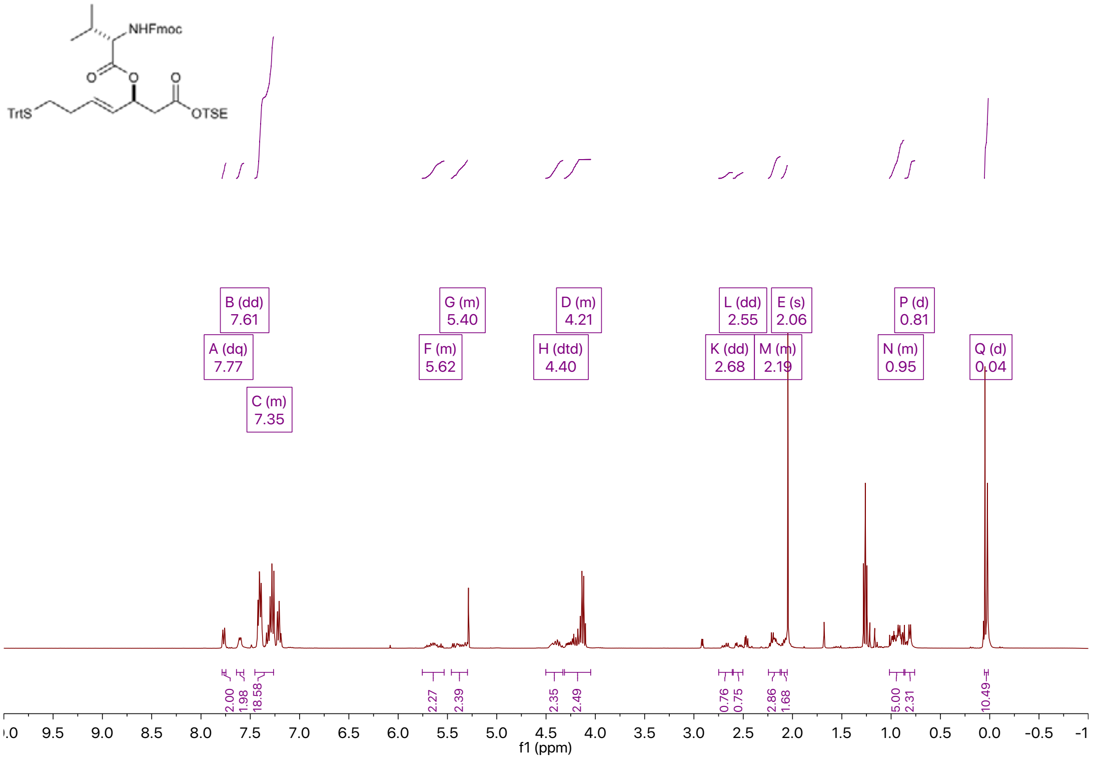

^{1}H NMR of **ABC1**

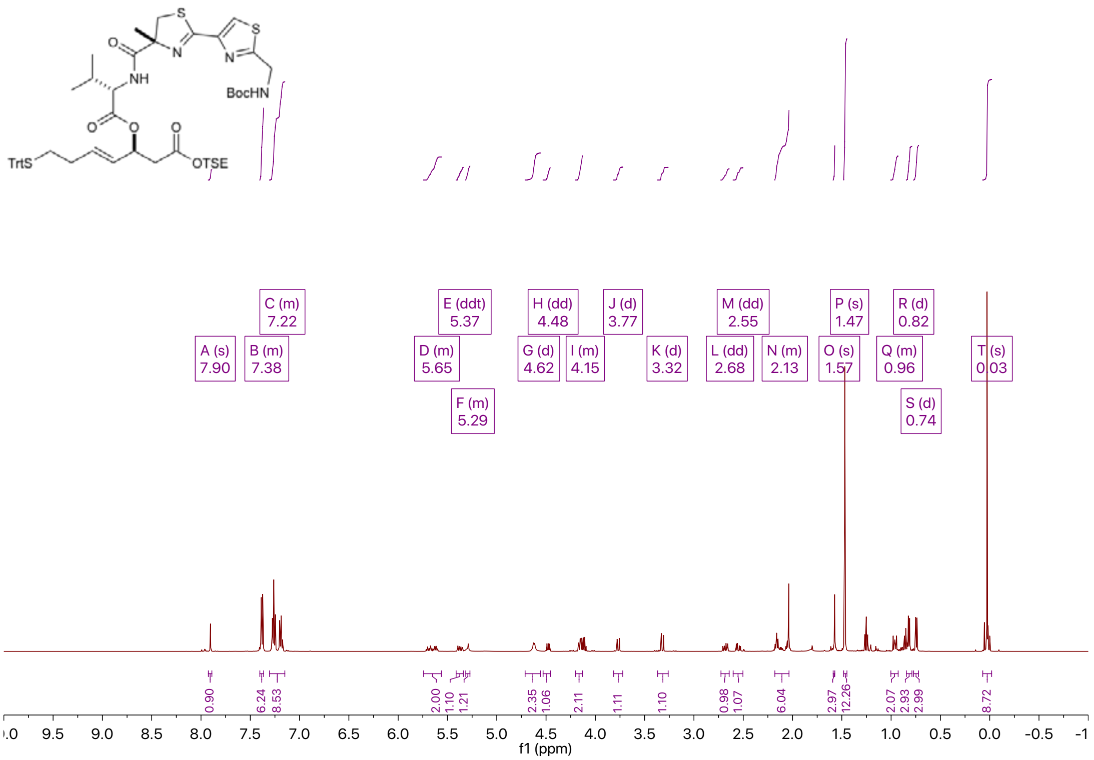

^{13}C NMR of **ABC1**

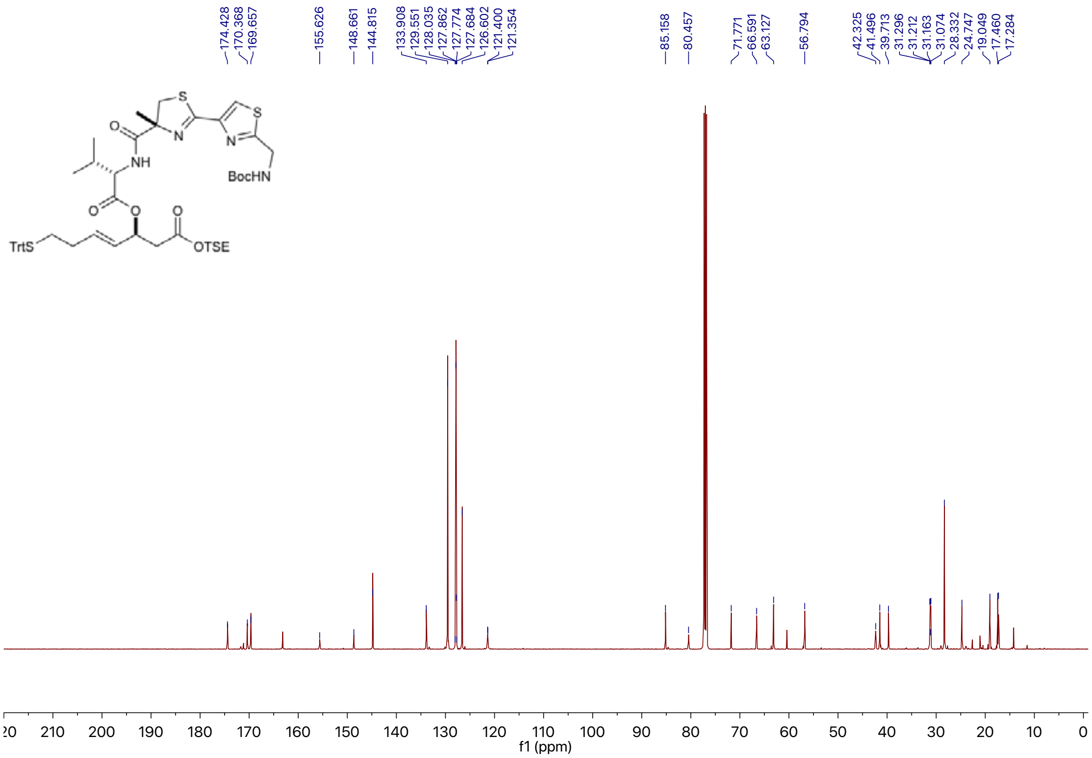

^{1}H NMR of **ABC3**

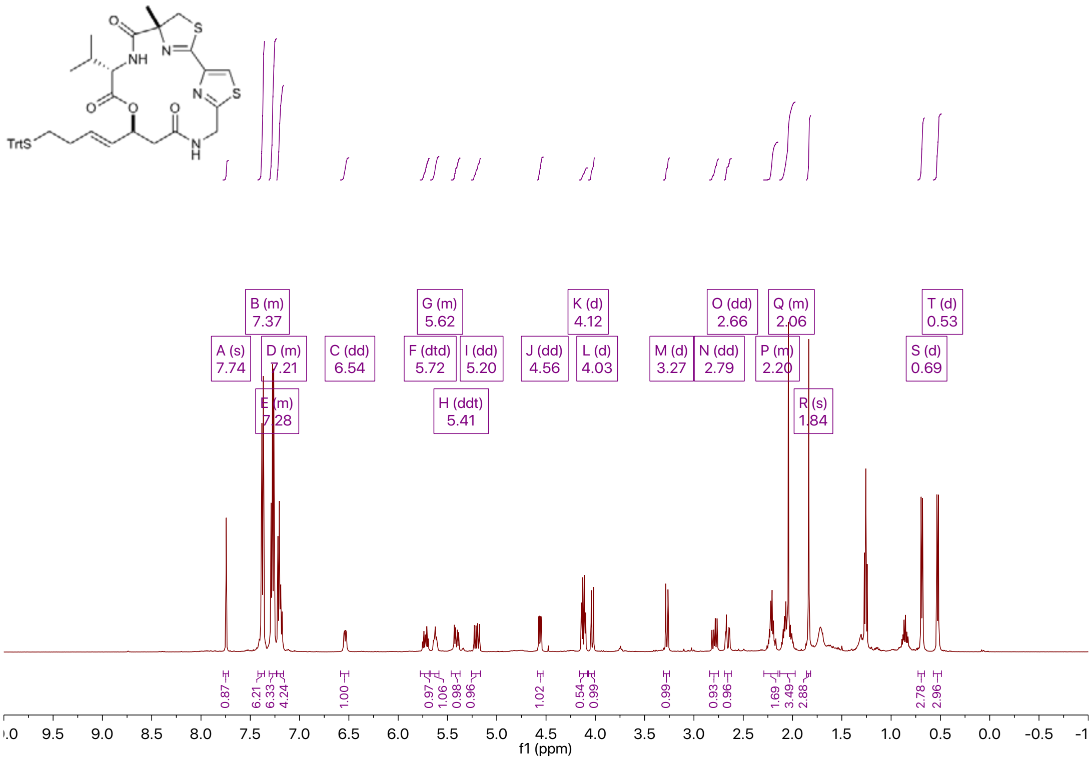

^{13}C NMR of **ABC3**

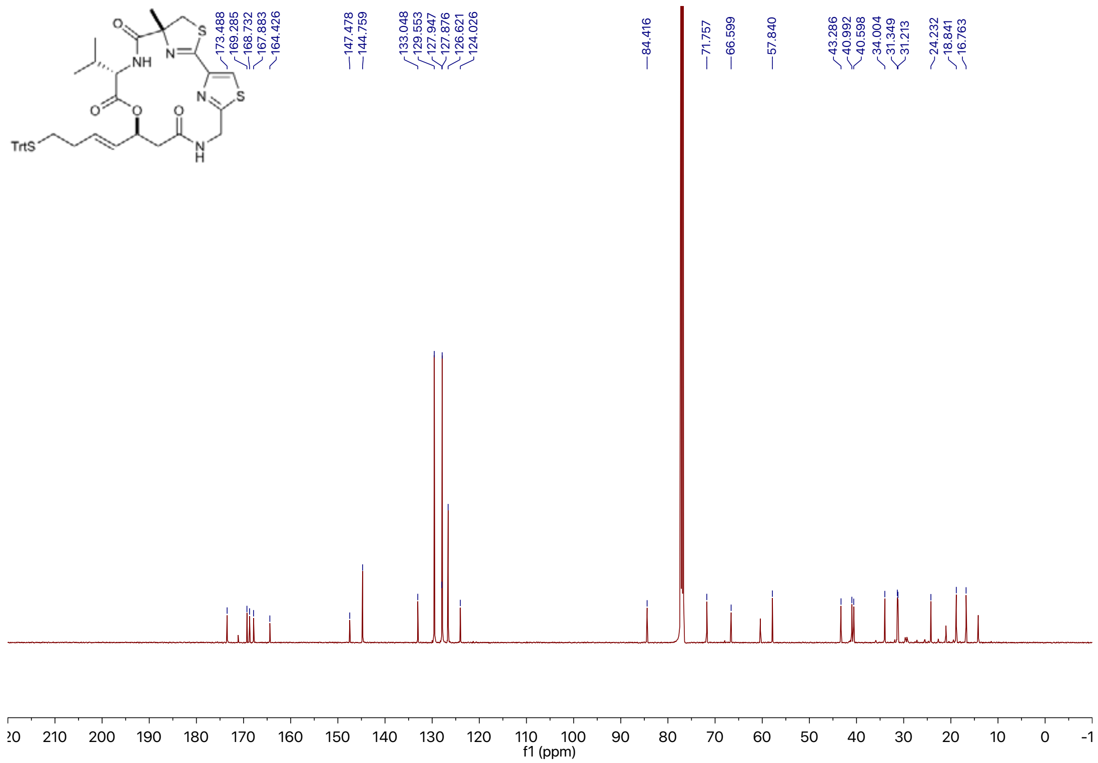

^{1}H NMR of **Largazole Thiol**

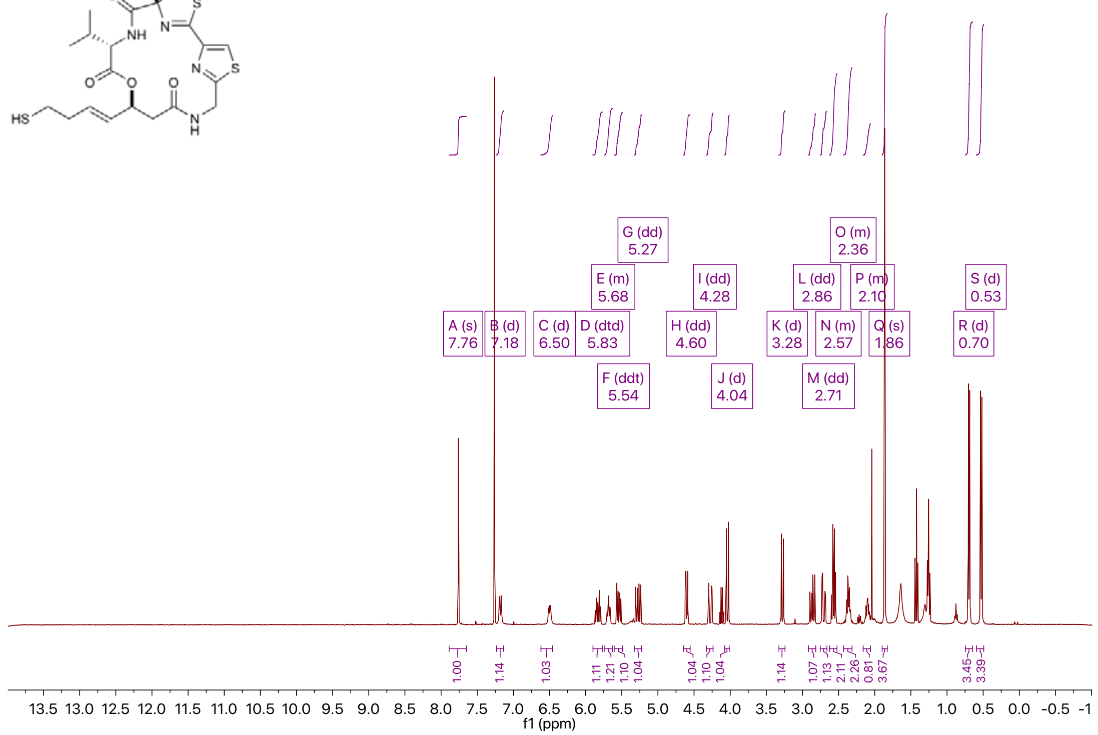

^{1}H NMR of **Largazole**

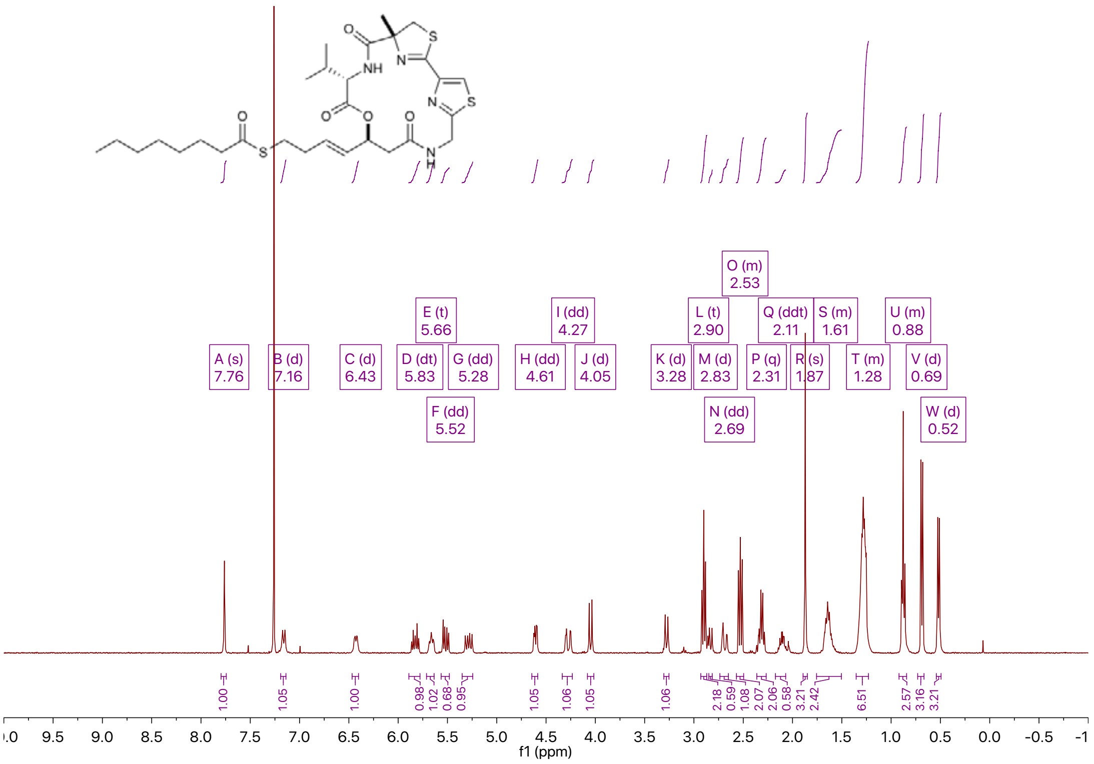

^{1}H NMR of **Stapled Largazole**

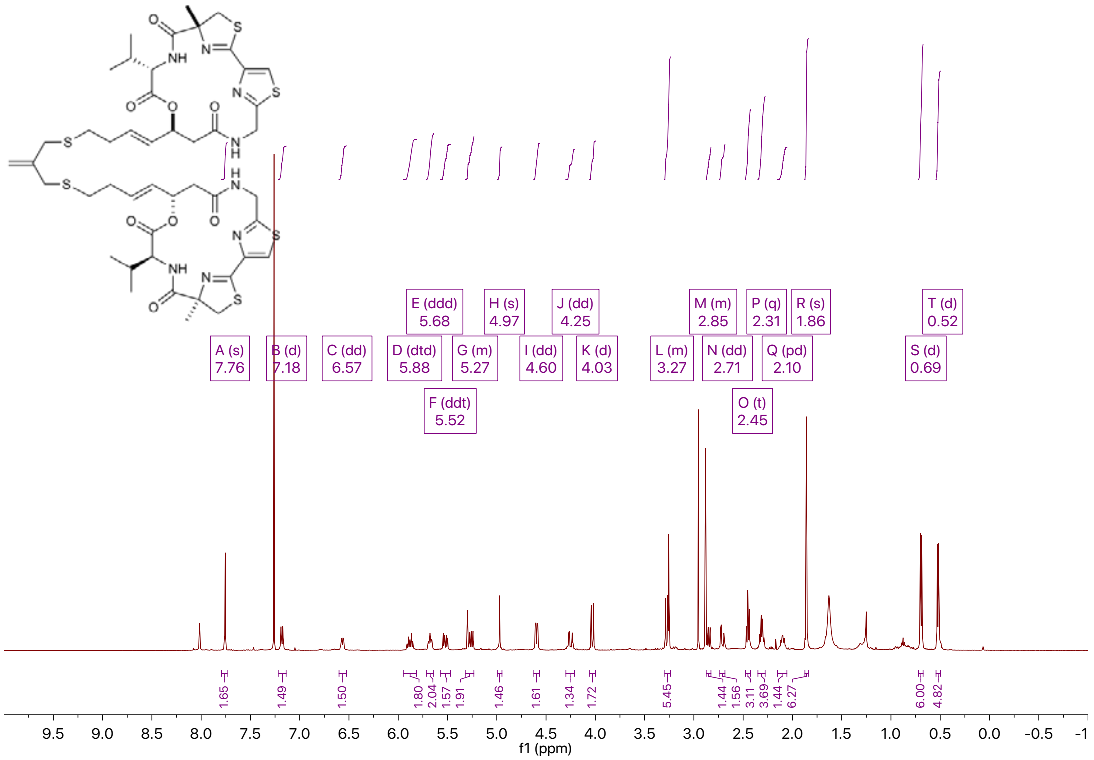

^{13}C NMR of **Stapled Largazole**

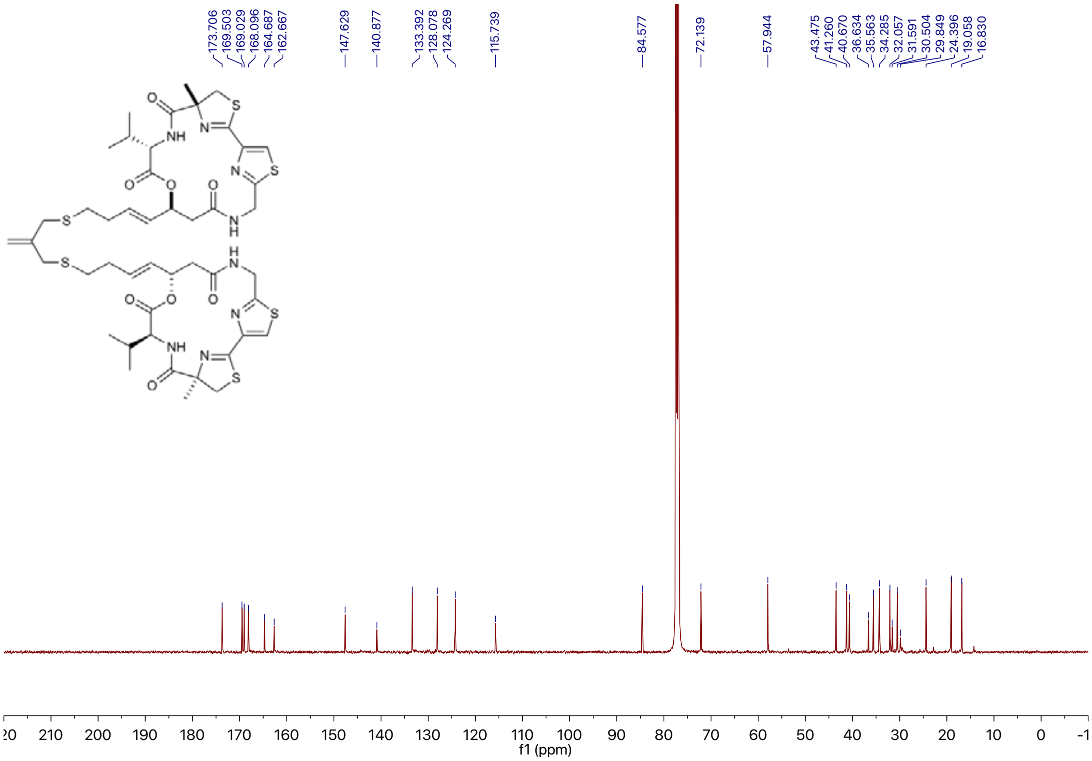

References

[1] S. Sun, I. Companon, N. Martinez-Saez, J. D. Seixas, O. Boutureira, F. Corzana, G. J. L. Bernardes, *ChemBioChem* **2018**, *19*, 48-52.

[2] A. R. Healy, M. I. Vizcaino, J. M. Crawford, S. B. Herzon, *J. Am. Chem. Soc.* **2016**, *138*, 5426-5432.

[3] A. Bowers, N. West, J. Taunton, S. L. Schreiber, J. E. Bradner, R. M. Williams, *J. Am. Chem. Soc.* **2008**, *130*, 11219-11222.

[4] B. Kitir, M. Baldry, H. Ingmer, C. A. Olsen, *Tetrahedron* **2014**, *70*, 7721-7732.

[5] P. Bhansali, C. L. Hanigan, R. A. Casero, L. M. Tillekeratne, *J. Med. Chem.* **2011**, *54*, 7453-7463.

[6] aD. J. Clausen, W. B. Smith, B. E. Haines, O. Wiest, J. E. Bradner, R. M. Williams, *Bioorg Med Chem* **2015**, *23*, 5061-5074; bS. Wen, G. Packham, A. Ganesan, *J. Org. Chem.* **2008**, *73*, 9353-9361.

[7] H. Benelkebir, S. Marie, A. L. Hayden, J. Lyle, P. M. Loadman, S. J. Crabb, G. Packham, A. Ganesan, *Bioorg. Med. Chem.* **2011**, *19*, 3650-3658.

[8] M. J. Frisch, G. W. Trucks, H. B. Schlegel, G. E. Scuseria, M. A. Robb, J. R. Cheeseman, G. Scalmani, V. Barone, G. A. Petersson, H. Nakatsuji, X. Li, M. Caricato, A. V. Marenich, J. Bloino, B. G. Janesko, R. Gomperts, B. Mennucci, H. P. Hratchian, J. V. Ortiz, A. F. Izmaylov, J. L. Sonnenberg, Williams, F. Ding, F. Lipparini, F. Egidi, J. Goings, B. Peng, A. Petrone, T.

Henderson, D. Ranasinghe, V. G. Zakrzewski, J. Gao, N. Rega, G. Zheng, W. Liang, M. Hada, M. Ehara, K. Toyota, R. Fukuda, J. Hasegawa, M. Ishida, T. Nakajima, Y. Honda, O. Kitao, H. Nakai, T. Vreven, K. Throssell, J. A. Montgomery Jr., J. E. Peralta, F. Ogliaro, M. J. Bearpark, J. J. Heyd, E. N. Brothers, K. N. Kudin, V. N. Staroverov, T. A. Keith, R. Kobayashi, J. Normand, K. Raghavachari, A. P. Rendell, J. C. Burant, S. S. Iyengar, J. Tomasi, M. Cossi, J. M. Millam, M. Klene, C. Adamo, R. Cammi, J. W. Ochterski, R. L. Martin, K. Morokuma, O. Farkas, J. B. Foresman, D. J. Fox, Revision B.01 ed., Gaussian, Inc., Wallingford CT, **2016**.

[9] Y. Zhao, D. G. Truhlar, *Theor. Chem. Acc.* **2008**, *120*, 215-241.

[10] G. Scalmani, M. J. Frisch, *J. Chem. Phys.* **2010**, *132*.

[11] R. F. Ribeiro, A. V. Marenich, C. J. Cramer, D. G. Truhlar, *J. Phys. Chem. B* **2011**, *115*, 14556-14562.

[12] aC. Gonzalez, H. B. Schlegel, *J. Chem. Phys.* **1989**, *90*, 2154-2161; bC. Gonzalez, H. B. Schlegel, *J. Phys. Chem.* **1990**, *94*, 5523-5527.
